# Supplementary material for: Spatiotemporal Dynamics of Non‐Ecological Speciation in Rubyspot Damselflies (Hetaerina spp.)
Source: Mol Ecol. 2025 May 15;34(12):e17797. doi: 10.1111/mec.17797 (PMC12143376; doi:10.1111/mec.17797)
Supplement: Supplementary file 1 — Data S1 [file MEC-34-e17797-s001.pdf]

Supporting Information for Spatiotemporal dynamics of non-ecological speciation in rubyspot damselflies (*Hetaerina* spp.)

Patterson, C., Brennan, A., Cowling, H., González-Rodríguez, A., Grether, G.F., Mendoza Cuenca, L. Springer, M., Vega-Sánchez, Y. M., and Drury, J.

Supplementary Methods
Supplementary Tables 1-5
Supplementary Figures 1-26

Supplementary Methods

Library preparation was conducted twice creating two separate ddRAD libraries. Library One was constructed at the NERC environmental omics facility (NEOF), at the University of Sheffield, using the ddRAD protocol from DaCosta and Sorenson (2014). Library Two was conducted at Durham University following the methodology from Franchini *et al.* (2017). Samples extracted for ddRAD library One were initially resuspended using 200µl of buffer AE and then were desiccated and resuspended in 50µl of buffer AE. Samples extracted for ddRAD library Two were resuspended in 50µl of buffer AE. DNA degradation was visualised via gel electrophoresis and the concentration calculated on a Qubit 2.0 Fluorometer broad range (BR). DNA concentration ranged from 0.47 ng/µl to 26.8 ng/µl with a mean of 4.44 ng/µl. For each sample, this totalled a range 5361ng to 95.4ng of DNA with an average of 888.32ng.

Library One

Double digest restriction enzyme association DNA (ddRAD) was created for 190 *Hetaerina* samples (183 *H. titia*, 4 *H. occisa*, and 3 *H. americana* senso lato) using two, six-base restriction enzymes, PstI and EcoRI. Library preparation was conducted using custom Illumina adapters; 92 barcoded P1 adapters and two P2 adapters with 192 unique barcode combinations. P2 adapters contained 8 bases of random nucleotides for the detection of PCR duplication (DaCosta & Sorenson, 2014).

Final DNA concentration for each of the 16 sample pools was calculated by fluorometer and ranged from 84.4ng/µl to 2.99ng/µl, with an average of 30.1ng/µl. 40ng of 16 samples were pooled into separate libraries and then quantified by qPCR. Separate libraries were then pooled into a single library for sequencing and sent for paired end 150 bp sequencing on Illumina NovaSeq 6000.

Bioinformatics for Library One

Sequencing was conducted on a NovaSeq 6000 (Illumina) within a single lane. Raw sequence files were demultiplex into one of the two P2 adapters (BC1 and BC2) and processed separately. Initially 212.1x10^6 BC1 paired read files and 189.1x10^6 BC2 paired read files were identified. Trimming was conducted using *trimomatic 0.39* to remove low quality bases using a sliding window of 4 and an average base quality of 20. Reads shorter than 15 bases were removed. After filtering 169.9 x10^6 (80.1%) BC1 paired sequences and 153.6x10^6 (81.2%) BC2 paired sequences remained.

Sequences were processed with STACKS v2.60 (Catchen et al., 2013). clone_filter identified 15.12% of BC1 reads to be PCR clones and were removed, retaining 144.2x10^6 paired reads. pcr_filter identified 19.66% of BC2 reads to be PCR clones and removed retaining 123.4x10^6 paired reads.

Reads were demultiplexed using process_radtags using the 8 base unique barcodes on the P1 adapters. 268,996,468 (93.3%) of BC1 reads were retained and assigned to a specific sample, 6.4% of reads did not contain an identifiable barcode and 0.3% could not identify the RAD cut site. 229,259,030 (92.9%) of BC2 reads were retained and assigned to a specific sample, 6.8% did not contain an identifiable barcode and 0.4% could not identify a RAD cut site. All remaining sequence were processed through *cutadapt* to remove both the remaining adapter regions. For all samples the total number of identified paired sequences was 496,388,354. The mean number of reads per sample was 1,292,678 (sd = 824,724) and ranged from 256 to 4,174,260.

Library Two

Double digest restriction enzyme association DNA (ddRAD) was created for 192 *Hetaerina* samples (112 *H. titia*, 80 *H. americana* senco lato) again using restriction enzymes PstI and EcoRI. Library preparation was done using custom Illumina adapters; three inner i5 barcoded adapters and four inner i7 adapters plus four outer i5 primers and four outer i7 primers creating 192 unique barcode combinations. Both inner i5 and i7 adapters contained a sequence of 4 bases of random nucleotides for the detection of PCR duplication. This protocol follows Franchini et al. (2017) but the adapters were modified to ligate to PstI and EcoRI restriction sites.

Final size selection was conducted using a pippin prep 2% agarose gel cassette with DF Marker L. 30µl of cleaned pooled PCR product was loaded onto a single lane and size selected for 250-400bp. 40µl of size selected digested, ligated, and indexed DNA was retrieved from the pippin prep elusion module, DNA concentration was quantified using the qubit high sensitivity and size range was quantified using a Tapestation HS d1000 cassette. This yield 40µl of quaddRAD library at 1.08ng/µl (43.2ng in total) which was sent for paired end 150 bp sequencing on Illumina NovaSeq 6000.

Bioinformatics for Library Two

Sequencing was conducted on a NovaSeq 6000 (Illumina) within a single lane. Raw sequence files were demultiplex into one of 16 combinations of outer barcodes. Each pool contained on average 16.1×10^6 paired reads (range 7.3×10^6 to 16.1×10^6) with 294.1×10^6 paired reads in total. Trimming was conducted using *trimomatic 0.39* to remove low quality bases using a sliding window of 4 and an average base quality of 20. Reads shorter than 15 bases were removed. After filtering, 289.5×10^6 (98.2%) sequences remained. Sequences were processed with *STACKS v2.60* (Catchen et al., 2013). *clone_filter* identified 17.4% of reads to be PCR clones and were removed, retaining 239.3×10^6 paired reads.

Sequences were demultiplexed using *process_radtags* which generated 192 individual samples files. In total 215.5×10^6 paired reads (90.1%) were assigned to a specific sample ranging from 671 to 13.3×10^6 (mean = 1.12×10^6) reads per sample.

SNP and Loci calling

For all SNP libraries*, samtools v1.13* was used to align all demultiplex sequence data and stored as a compressed sequence alignment file (.bam). After aligning the sequence reads to the draft genomes, we excluded samples that had a mean depth of less than 5.

Genotype calling was done using *bcftools v1.13* (Danecek et al., 2021; Li, 2011) using the *mpileup* and *call* commands, with a max depth of 10,000 and a prior expected substitution rate of 1e-6. As our samples were collected over a large geographical scale and included multiple species, we did not use Hardy-Weinberg equilibrium calculations for variant calling. After genotyping, variant sites were restricted to SNPs. SNP sites were filtered to remove all SNP calls with a read depth of less than 10, an average read depth across samples greater than 200 per sample, and a quality score less than 20. Further filtering removed SNPs that had a minor allele frequency less than 0.05% (<2 samples), SNPs that were found in less than 80% of samples, and to avoid linkage we randomly selected one SNP from a window of 1000 base pairs using the *prune+* command in *bcftools* (Danecek et al., 2021). See break down in Supplementary Table 1)

De novo SNP calling using ipyrad

There are large phylogenetic distances (> 6 million years) between and within the species used in our study (Standring et al., 2022; Vega-Sánchez et al., 2024). Mapping reads to draft genomes with high phylogenetic distance to the populations and species within a

study can lead to ascertainment bias in SNP calling (Bohling, 2020). As such we used ipyrad (Eaton & Overcast, 2020) to assembly RAD loci de novo. To reduce computation time, we used a subset of the top three highest coverage samples from each lineage identified using the SNP libraries constructed using draft genomes (18 samples in total across six lineages). The assignment of samples to each lineage did not change across any SNP library. We ran ipyrad following default parameters of the manual. *Denovo* mapping of the sequenced read identified 42,109 RAD loci, with each sample having an average of 14,693 RAD loci sequenced (range 10,319 to 17,554). SNPs were filtered to one random SNP per RAD loci and then removed loci that were missing from more than 20% of samples. For de novo SNP libraries that contained both *H. americana* sensu lato and *H. titia* retained a data set of 518 SNPs. The SNP library that contained only *H. americana* sensu lato retained 2,652 SNPs. The SNP library that contained only *H. titia* retained 1,733.

Re-sequencing of highly heterozygous samples

Pre-liminary analysis, using the r package SambaR (de Jong et al., 2021), identified five samples as having high levels of heterozygosity - one sample from Library One and four samples from Library Two. The high heterozygosity samples from Library Two were processed within the same pool and had the same i5 inner barcode and are likely due to a fault in inner barcode ligation. The likely cause of the high heterozygosity for the sample from Library One could not be determined. To determine whether laboratory error was the cause for the high heterozygosity, we re-extracted DNA (from tissue) for the five identified samples and re-sequenced them in line with the methodology described in Library Two. We attained, 4.7×10^6 paired reads of sequence data which was trimmed to 4.6×10^6 paired reads, removed clones retaining 4.2×10^6 paired reads, and demultiplexed which retained 4.1×10^6 paired reads using the methodology described in Library Two. All four samples from Library Two did not show high levels of heterozygosity after re-sequencing. The sample from Library One retained high levels of heterozygosity.

Migration analyses in delimitR

To decrease computation time while running *delimitR* (Smith & Carstens, 2020), we wrote a custom R function called *fastsimcoalsim_sbatch*. Built off the default *delimitR* function *fastsimcoalsim*, *fastsimcoalsim_sbatch* allows each set of fastsimcoal simulations (for each demographic scenario) to be run in parallel using the SLURM HPC management system.

We simulated each scenario using broad, uniform priors. Effective populations size (N*e*) was set from 100,000 to 2,000,000 haploid individuals (Ne/2), divergence time for the first population split (Tdiv1) was 500,000 to 12,000,000 generations, and divergence for the second population split (Tdiv2) was 500,000 to 8,000,000 generations. We set Tdiv1 to be always greater Tdiv2. Migration rates were set from 0.000005 to 0.0005 as a proportion of N*e* per generation.

Historical demography analyses in G-PhoCS

In comparison to previous analysis which used SNP datasets, G-PhoCS uses sequences of whole loci. G-PhoCS uses MCMCcoal to estimate divergence times and effective population sizes from multilocus sequence data and allows users to specify migration bands between lineages.

For the SNP mapped to the draft genomes, we used a custom R script to generate consensus sequences for each whole ddRAD loci for each sample using the bcftools consensus command. We identified the position of ddRAD loci using the position of the SNPs identified during the previous calling pipeline. We generated consensus sequences, for

each sample, 500 base pairs either side of each identified SNPs. We trimmed loci to remove bases that were outside of the sequenced region then removed loci that had a sequence length of less than 100bp and had greater than 20% missing bases. For the de novo SNP library we generated the RAD loci sequence file using the gphocs output option within ipyrad.

Due to computational constraints, we restricted G-PhoCS analysis to the four samples with highest loci coverage from each ancestral cluster identified by sNMF and include no more than 2000 randomly selected ddRAD loci, similar to Prates *et al.*, (2018). We used the tree topology generated from RAxML, SVDquartet, and SNAPP and ran G-PhoCS with and without migration for all possible migration bands within the tree (with the exception of migration from either of the recently diverged lineages to the older lineage, which is an unresolved issue within G-PhoCS: https://github.com/gphocs-dev/G-PhoCS/issues/25). We ran G-PhoCS with a range of uninformative priors following Prates *et al* (2018). The effective population size (theta - θ) and the divergence times (tau - τ) priors were set using gamma distributions of α=1 and β=20, and α=1 and β=200, respectively. The migration rate prior was set to a gamma distribution with α=1 and β=2e-7. To avoid ascertainment bias in loci mapped to a more closely related draft genome we compared divergence times between lineages of *H. titia* and *H. americana* using loci mapped to the relative heterospecific draft genome and compared that with the de novo loci.

Each run of G-PhoCS was conducted for 1,000,000 iterations with a 10% burn-in and sampled every 100 iterations. We optimised the initial acceptance rate to be 30-40% by running 100 interactions of automatic fine-tuning. Convergence was confirmed by visualising trace plots in R. The R package *coda* was used to calculate the effective sample size (ESS) and the 95% Highest Posterior Density (HPD) from the trace file. We converted mutation rate-scaled parameter estimates of G-PhoCS into the number of diploid individuals and the number of years using 2.8e-9 mutations per base pair per generation (Keightley et al., 2014). We converted generations to years using an estimated generation time of one year.

Supplementary Table 1: The total number of variants identified in calling and the number of variants remaining after the following filtering steps. Removing indels (All single base calls). Removing all base calls that had read depth of less than 10 and greater than 200, and a quality score less than 20 (DP10-200, Q>20). Removing all SNPs not genotyped for 80% of samples, removing SNPs with a minimum allele frequency less than 0.05, and randomly removing all but one SNP within 1000 base window.

Species	Draft genome	Number of samples	All variant calls	All single base calls	DP10-200, Q>20	80% Samples	MAF>0.05	Random 1-1000
all	*H. americana*	364	134810308	134707986	2233411	80344	34506	1156
all	*H. titia*	363	161441042	161305711	2871466	81701	36376	1199
H. americana/ calverti	*H. americana*	75	73615352	73549147	4842870	1042635	151033	6997
H. americana/ calverti	*H. titia*	75	44189192	44164151	2258691	495629	80935	4103
H. titia	*H. americana*	286	81630947	81586704	1709783	260765	73471	3674
H. titia	*H. titia*	285	136427605	136309637	2997868	494012	121745	6369

Supplementary Table 2: Overview of all analysis presented within the paper and which SNP/loci libraries were used. Each library consists of different combinations of samples from different species and reads were aligned to the draft genome of *Hetaerina americana* (Grether et al., 2023), *Hetaerina titia* (Patterson et al., 2023) or mapped de novo. Each analysis and library are marked with whether the results are presented in the main text (M) or in the supplementary (S). Table includes the number of samples (N) and the total number of SNPs/loci that were included in analysis.

			**Population structure**			**Phylogentics**						**Demographic/ divergence times**									**Introgression**		
			**sNMF & PCA**			**RAxML**			**SVDQuartets**			**delimitR**			**SNAPP**			**G-Phocs**			**Introgress**		
**Library Number**	**Included species**	**Read alignment**	**SNPs**	**N**	**M/S**	**SNPs**	**N**	**M/S**	**SNPs**	**N**	**M/S**	**2N**	**Down Sample (2N)**	**M/S**	**SNPs**	**N**	**M/S**	**Loci**	**N**	**M/S**	**SNPs (sex-linked)**	**N**	**M/S**
#1	*H. americana* sensu lato & *H. titia*	HetAmer1.0	-	-	-	524	258	S	566	258	S	-	-	-	571	98	**M**	-	-	-	-	-	-
#2	*H. americana* sensu lato & *H. titia*	HetTit1.0	-	-	-	560	254	S	609	254	S	-	-	-	603	95	**M**	-	-	-	-	-	-
#3	*H. americana* sensu lato & *H. titia*	*de novo*	-	-	-	-	-	-	-	-	-	-	-	-	519	18	**M**	-	-	-	-	-	-
#4	*H. americana* sensu lato	HetAmer1.0	5259	58	**M**	3949	58	**M**	5259	58	S	22,20,72	10,12,18	S	-	-	-	2000	9	S	-	-	-
#5	*H. americana* sensu lato	HetTit1.0	1816	58	S	1034	58	S	1816	58	S	22,20,72	10,11,18	S	-	-	-	1814	9	S	-	-	-
#6	*H. americana* sensu lato	*de novo*	-	-	-	-	-	-	-	-	-	-	-	-	-	-	-	2652	9	**M**	-	-	-
#7	*H. titia*	HetAmer1.0	1122	207	S	846	202	S	1122	202	S	100,204,92	20,25,20	S	-	-	-	1122	9	S	-	-	-
#8	*H. titia*	HetTit1.0	3819	205	**M**	3020	199	**M**	3819	199	S	100,204,92	26,50,20	S	-	-	-	2000	9	S	914 (19)	33	**M**
#9	*H. titia*	*de novo*	-	-	-	-	-	-	-	-	-	-	-	-	-	-	-	1733	9	**M**	-	-	-

Supplementary Table 3: Geographical location of all samples for which adequate sequence data was attained. Species refers to the taxonomic classification assigned to sample upon collection, either *H. titia* or *H. americana* sensu lato. Collection date provided as YYYY-MM-DD. Lineage assignment refers to the lineage that the sample was assigned to after genotyping. Either *H. titia* Pacific (titia-Pac), *H. titia* North Atlantic (titia-NAtl), *H. titia* South Atlantic (titia-SAtl), *H. calverti* (calverti), *H. americana* South (americana-S) or *H. americana* North (americana-N).

Sample Name	Sex	Species	Site ID	Lat	Long	Collection Date	Country	Province	Ocean drainage	Lineage assignment
**10OT23**		titia	10OT	18.68	-96.38	2010-08-08	Mexico	Veracruz	Gulf	titia-SAtl
**11CT10**	M	titia	11CT	18.37	-95	2011-07-04	Mexico	Veracruz	Gulf	titia-SAtl
**11CT12**		titia	11CT	18.37	-95	2011-07-04	Mexico	Veracruz	Gulf	titia-SAtl
**11CT18**	M	titia	CT	18.37	-95	2011-07-04	Mexico	Veracruz	Gulf	titia-SAtl
**11CT25**	F	titia	CT	18.37	-95	2011-07-04	Mexico	Veracruz	Gulf	titia-SAtl
**ACSFa01**	M	titia	ACSF01	29.83	-82.65	2019-07-10	United States	Florida	Gulf	titia-NAtl
**ACSFa02**	M	titia	ACSF01	29.83	-82.65	2019-07-10	United States	Florida	Gulf	titia-NAtl
**ACSFa03**	M	titia	ACSF01	29.83	-82.65	2019-07-10	United States	Florida	Gulf	titia-NAtl
**ACSFc01**	M	titia	ACSF03	29.83	-82.68	2019-07-10	United States	Florida	Gulf	titia-NAtl
**ACSFc02**	M	titia	ACSF03	29.83	-82.68	2019-07-10	United States	Florida	Gulf	titia-NAtl
**ACSFc03**	M	titia	ACSF03	29.83	-82.68	2019-07-10	United States	Florida	Gulf	titia-NAtl
**ACTPa02**	M	titia	ACTP01	15.29	-92.67	2021-06-18	Mexico	Chiapas	Pacific	titia-Pac
**ACTPa03**	M	titia	ACTP01	15.29	-92.67	2021-06-18	Mexico	Chiapas	Pacific	titia-Pac
**ACTPa08**	M	titia	ACTP01	15.29	-92.67	2021-06-18	Mexico	Chiapas	Pacific	titia-Pac
**AR0101**	M	titia	AR01	33.9	-92.23	2019-08-19	United States	Arkansas	Gulf	titia-NAtl
**AR0102**	M	titia	AR01	33.9	-92.23	2019-08-19	United States	Arkansas	Gulf	titia-NAtl

Sample Name	Sex	Species	Site ID	Lat	Long	Collection Date	Country	Province	Ocean drainage	Lineage assignment
**AR0103**	M	titia	AR01	33.9	-92.23	2019-08-19	United States	Arkansas	Gulf	titia-NAtl
**AR0104**	M	titia	AR01	33.9	-92.23	2019-08-19	United States	Arkansas	Gulf	titia-NAtl
**AR0207**	M	titia	AR02	33.7	-92.03	2019-08-20	United States	Arkansas	Gulf	titia-NAtl
**AR0208**	M	titia	AR02	33.7	-92.03	2019-08-20	United States	Arkansas	Gulf	titia-NAtl
**AR0209**	M	titia	AR02	33.7	-92.03	2019-08-20	United States	Arkansas	Gulf	titia-NAtl
**AR0210**	M	titia	AR02	33.7	-92.03	2019-08-20	United States	Arkansas	Gulf	titia-NAtl
**ARa02**	M	titia	AR	18.95	-103.95	2005-07-26	Mexico	Colima	Pacific	titia-Pac
**ARa04**	F	titia	AR	18.95	-103.95	2005-07-16	Mexico	Colima	Pacific	titia-Pac
**ARa05**	M	titia	AR	18.95	-103.95	2005-07-16	Mexico	Colima	Pacific	titia-Pac
**BALB01**	M	titia	BALB	10.05	-83.33	2017-03-31	Costa Rica	Limón	Caribbean	titia-SAtl
**BALB02**	M	titia	BALB	10.05	-83.33	2017-03-31	Costa Rica	Limón	Caribbean	titia-SAtl
**BALB06**	M	titia	BALB	10.05	-83.33	2017-03-31	Costa Rica	Limón	Caribbean	titia-SAtl
**BAMB03**	M	titia	BAMB	10.06	-83.37	2017-03-31	Costa Rica	Limón	Caribbean	titia-SAtl
**BAMB06**	M	titia	BAMB	10.06	-83.37	2017-03-31	Costa Rica	Limón	Caribbean	titia-SAtl
**BZ1509**	M	titia	BZ15	17.01	-88.48	2021-06-17	Belize		Caribbean	titia-SAtl
**BZ1513**	M	titia	BZ15	17.01	-88.48	2021-06-17	Belize		Caribbean	titia-SAtl
**BZ2105**	M	titia	BZ21	17.17	-89.11	2021-06-21	Belize		Caribbean	titia-SAtl
**BZ2106**	M	titia	BZ21	17.17	-89.11	2021-06-21	Belize		Caribbean	titia-SAtl
**BZ2303**	M	titia	BZ23	17.15	-88.71	2021-06-17	Belize		Caribbean	titia-SAtl
**BZ2601**	M	titia	BZ26	17.26	-88.79	2021-06-17	Belize		Caribbean	titia-SAtl
**BZ2604**	M	titia	BZ26	17.26	-88.79	2021-06-17	Belize		Caribbean	titia-SAtl
**BZ3004**	M	titia	BZ30	17.56	-88.52	2021-06-18	Belize		Caribbean	titia-SAtl
**BZ3005**	M	titia	BZ30	17.56	-88.52	2021-06-18	Belize		Caribbean	titia-SAtl

Sample Name	Sex	Species	Site ID	Lat	Long	Collection Date	Country	Province	Ocean drainage	Lineage assignment
**BZ3204**	M	titia	BZ32	17.9	-88.85	2021-06-19	Belize		Caribbean	titia-SAtl
**BZ3205**	M	titia	BZ32	17.9	-88.85	2021-06-19	Belize		Caribbean	titia-SAtl
**BZ3209**	F	titia	BZ32	17.9	-88.85	2021-06-19	Belize		Caribbean	titia-SAtl
**BZ4001**	M	titia	BZ40	17.11	-88.66	2021-06-17	Belize		Caribbean	titia-SAtl
**BZ4002**	M	titia	BZ40	17.11	-88.66	2021-06-17	Belize		Caribbean	titia-SAtl
**BZ4602**	M	titia	BZ46	16.86	-89.04	2021-06-22	Belize		Caribbean	titia-SAtl
**BZ4603**	M	titia	BZ46	16.86	-89.04	2021-06-22	Belize		Caribbean	titia-SAtl
**BZ4801**	M	titia	BZ48	16.67	-88.45	2021-06-27	Belize		Caribbean	titia-SAtl
**BZ4802**	M	titia	BZ48	16.67	-88.45	2021-06-27	Belize		Caribbean	titia-SAtl
**BZ4905**	M	titia	BZ49	16.51	-88.48	2021-06-27	Belize		Caribbean	titia-SAtl
**BZ5001**	M	titia	BZ50	16.45	-88.74	2021-06-27	Belize		Caribbean	titia-SAtl
**BZ5005**	M	titia	BZ50	16.45	-88.74	2021-06-27	Belize		Caribbean	titia-SAtl
**BZ5206**	M	titia	BZ52	16.15	-89.01	2021-06-27	Belize		Caribbean	titia-SAtl
**CA0103**	M	titia	CA01_CR	9.76	-84.62	2014-05-13	Costa Rica	Puntarenas	Pacific	titia-Pac
**CA0104**	M	titia	CA01_CR	9.76	-84.62	2014-05-13	Costa Rica	Puntarenas	Pacific	titia-Pac
**CUAJa01**	M	titia	CUAJ01	16.79	-95.01	2021-06-22	Mexico	Oaxaca	Gulf	titia-SAtl
**CUAJa02**	M	titia	CUAJ01	16.79	-95.01	2021-06-22	Mexico	Oaxaca	Gulf	F1 (titia-SAtl and titia-Pac)
**CUAJa04**	M	titia	CUAJ01	16.79	-95.01	2021-06-22	Mexico	Oaxaca	Gulf	titia-SAtl
**CUAJb04**	M	titia	CUAJ02	16.83	-95	2021-06-22	Mexico	Oaxaca	Gulf	titia-SAtl
**CUAJb05**	M	titia	CUAJ02	16.83	-95	2021-06-22	Mexico	Oaxaca	Gulf	titia-SAtl
**CVa02**	M	titia	CV	29.33	-98.87	2012-06-25	United States	Texas	Gulf	titia-NAtl
**CVa03**	M	titia	CV	29.33	-98.87	2012-06-25	United States	Texas	Gulf	titia-NAtl
**CVa04**	M	titia	CV	29.33	-98.87	2012-06-23	United States	Texas	Gulf	titia-NAtl

Sample Name	Sex	Species	Site ID	Lat	Long	Collection Date	Country	Province	Ocean drainage	Lineage assignment
**CVa08**	M	titia	CV	29.33	-98.87	2012-06-23	United States	Texas	Gulf	titia-NAtl
**CVa13**	M	titia	CV	29.33	-98.87	2012-06-23	United States	Texas	Gulf	titia-NAtl
**CYa04**	M	titia	CY	21.75	-98.96	2004-07-20	Mexico	San Luis Potosí	Gulf	titia-NAtl
**CYa07**	F	titia	CY	21.75	-98.96	2004-07-20	Mexico	San Luis Potosí	Gulf	titia-NAtl
**CYa10**	F	titia	CY	21.75	-98.96	2004-07-20	Mexico	San Luis Potosí	Gulf	titia-NAtl
**CYa11**	F	titia	CY	21.75	-98.96	2004-07-20	Mexico	San Luis Potosí	Gulf	titia-NAtl
**ESRBa10**	M	titia	ESRB1	9.72	-82.97	2017-04-29	Costa Rica	Limón	Caribbean	titia-SAtl
**GA0203**	M	titia	GA02	8.7	-83.19	2014-05-16	Costa Rica	Puntarenas	Pacific	titia-Pac
**GCJRa01**	M	titia	GCJR01	37.67	-77.89	2019-07-13	United States	Virginia	Atlantic	titia-NAtl
**GCJRa02**	M	titia	GCJR01	37.67	-77.89	2019-07-13	United States	Virginia	Atlantic	titia-NAtl
**GCJRa05**	M	titia	GCJR01	37.67	-77.89	2019-07-13	United States	Virginia	Atlantic	titia-NAtl
**GOa02**	M	titia	GO01	8.64	-83.2	2016-05-17	Costa Rica	Puntarenas	Pacific	titia-Pac
**GOa07**	M	titia	GO01	8.64	-83.2	2016-06-21	Costa Rica	Puntarenas	Pacific	titia-Pac
**HCARa02**	M	titia	HCAR01	27.88	-82.17	2019-07-08	United States	Florida	Gulf	titia-NAtl
**HCARa03**	F	titia	HCAR01	27.88	-82.17	2019-07-08	United States	Florida	Gulf	titia-NAtl
**HCARa05**	M	titia	HCAR01	27.88	-82.17	2019-07-08	United States	Florida	Gulf	titia-NAtl
**HCARb01**	M	titia	HCAR02	27.87	-82.14	2019-07-08	United States	Florida	Gulf	titia-NAtl
**HCARb02**	F	titia	HCAR02	27.87	-82.14	2019-07-08	United States	Florida	Gulf	titia-NAtl

Sample Name	Sex	Species	Site ID	Lat	Long	Collection Date	Country	Province	Ocean drainage	Lineage assignment
**HCICa01**	M	titia	HCIC01	28.09	-82.09	2019-07-09	United States	Florida	Gulf	titia-NAtl
**HCICa02**	M	titia	HCIC01	28.09	-82.09	2019-07-09	United States	Florida	Gulf	titia-NAtl
**HCICa03**	F	titia	HCIC01	28.09	-82.09	2019-07-09	United States	Florida	Gulf	titia-NAtl
**HtiCn13**	M	titia	HtiCn	18.25	-103.23	2014-06	Mexico	Michoacán	Pacific	titia-Pac
**HtiCn2**	M	titia	HtiCn	18.25	-103.23	2014-06	Mexico	Michoacán	Pacific	titia-Pac
**HtiCn7**	M	titia	HtiCn	18.25	-103.23	2014-06	Mexico	Michoacán	Pacific	titia-Pac
**HtiNR1**	M	titia	HtiNR	15.9	-91.99	2013-10	Mexico	Chiapas	Gulf	titia-SAtl
**HtiPo10**	M	titia	HtiPo	18.04	-103.54	2014-06	Mexico	Michoacán	Pacific	titia-Pac
**HtiPo2**	M	titia	HtiPo	18.04	-103.54	2014-06	Mexico	Michoacán	Pacific	titia-Pac
**HtiPo6**	M	titia	HtiPo	18.04	-103.54	2014-06	Mexico	Michoacán	Pacific	titia-Pac
**HtiTi14**	M	titia	HtiTi	18.67	-103.69	2014-06	Mexico	Michoacán	Pacific	titia-Pac
**HtiTi15**	M	titia	HtiTi	18.67	-103.69	2014-06	Mexico	Michoacán	Pacific	titia-Pac
**HXRCb05**	F	titia	HXRC02	15.75	-96.3	2021-06-08	Mexico	Oaxaca	Pacific	titia-Pac
**HXRCb06**	F	titia	HXRC02	15.75	-96.3	2021-06-08	Mexico	Oaxaca	Pacific	titia-Pac
**HXRCb08**	M	titia	HXRC02	15.75	-96.3	2019-08-09	Mexico	Oaxaca	Pacific	titia-Pac
**IA0406**	M	titia	IA04	41.3	-94.07	2019-08-09	United States	Iowa	Gulf	titia-NAtl
**IA0408**	M	titia	IA04	41.3	-94.07	2019-08-09	United States	Iowa	Gulf	titia-NAtl
**IA0413**	M	titia	IA04	41.3	-94.07	2019-08-09	United States	Iowa	Gulf	titia-NAtl
**IA0416**	F	titia	IA04	41.3	-94.07	2019-08-09	United States	Iowa	Gulf	titia-NAtl
**IA0417**	M	titia	IA04	41.3	-94.07	2019-08-09	United States	Iowa	Gulf	titia-NAtl
**IA0418**	M	titia	IA04	41.3	-94.07	2019-08-09	United States	Iowa	Gulf	titia-NAtl

**Sample Name**	**Sex**	**Species**	**Site ID**	**Lat**	**Long**	**Collection Date**	**Country**	**Province**	**Ocean drainage**	**Lineage assignment**
**IL0121**	M	titia	IL01	42.32	-89.36	2019-08-27	United States	Illinois	Gulf	titia-NAtl
**IL0123**	M	titia	IL01	42.32	-89.36	2019-08-27	United States	Illinois	Gulf	titia-NAtl
**IL0135**	M	titia	IL01	42.32	-89.36	2019-08-27	United States	Illinois	Gulf	titia-NAtl
**IL0140**	F	titia	IL01	42.32	-89.36	2019-08-27	United States	Illinois	Gulf	titia-NAtl
**Il0408**	M	titia	IL04	42.02	-89.33	2019-08-14	United States	Illinois	Gulf	titia-NAtl
**Il0409**	M	titia	IL04	42.02	-89.33	2019-08-14	United States	Illinois	Gulf	titia-NAtl
**IL0413**	M	titia	IL04	42.02	-89.33	2019-08-14	United States	Illinois	Gulf	titia-NAtl
**LCPCa02**	M	titia	LCPC1	31.14	-89.64	2017-07-06	United States	Mississippi	Gulf	titia-NAtl
**LCPCa03**	M	titia	LCPC1	31.14	-89.64	2017-07-06	United States	Mississippi	Gulf	titia-NAtl
**LCSBc01**	M	titia	LCSB3	31.16	-89.61	2017-07-06	United States	Mississippi	Gulf	titia-NAtl
**MACM02**	F	titia	MACM	10.1	-83.25	2017-04-01	Costa Rica	Limón	Caribbean	titia-SAtl
**MC02.001**	M	titia	MC02	30.49	-94.83	2012-06-18	United States	Texas	Gulf	titia-NAtl
**MC02.002**	M	titia	MC02	30.49	-94.83	2012-06-18	United States	Texas	Gulf	titia-NAtl
**MC02.003**	M	titia	MC02	30.49	-94.83	2012-06-18	United States	Texas	Gulf	titia-NAtl
**MEBA013928**	F	titia	MEBA01	29.72	-99.08	2021-08-16	United States	Texas	Gulf	titia-NAtl
**MI0111**	M	titia	MI01	42.23	-84.49	2019-08-28	United States	Michigan	Atlantic	titia-NAtl

Sample Name	Sex	Species	Site ID	Lat	Long	Collection Date	Country	Province	Ocean drainage	Lineage assignment
**MI0112**	M	titia	MI01	42.23	-84.49	2019-08-28	United States	Michigan	Atlantic	titia-NAtl
**MI0113**	M	titia	MI01	42.23	-84.49	2019-08-28	United States	Michigan	Atlantic	titia-NAtl
**MI0114**	F	titia	MI02	42.23	-84.49	2019-08-28	United States	Michigan	Atlantic	titia-NAtl
**MI0162**	F	titia	MI01	42.23	-84.49	2019-08-28	United States	Michigan	Atlantic	titia-NAtl
**MI0225**	F	titia	MI02	42.5	-83.74	2019-08-28	United States	Michigan	Atlantic	titia-NAtl
**MI0230**	F	titia	MI02	42.5	-83.74	2019-08-28	United States	Michigan	Atlantic	titia-NAtl
**MI0238**	M	titia	MI02	42.5	-83.74	2019-08-28	United States	Michigan	Atlantic	titia-NAtl
**MI0239**	M	titia	MI02	42.5	-83.74	2019-08-28	United States	Michigan	Atlantic	titia-NAtl
**MIXTa02**	M	titia	MIXT01	17.2	-95.21	2021-06-23	Mexico	Oaxaca	Gulf	titia-SAtl
**MIXTa04**	M	titia	MIXT01	17.2	-95.21	2021-06-23	Mexico	Oaxaca	Gulf	titia-SAtl
**MIXTa05**	M	titia	MIXT01	17.2	-95.21	2021-06-23	Mexico	Oaxaca	Gulf	titia-SAtl
**MIXTa10**	M	titia	MIXT01	17.2	-95.21	2021-06-23	Mexico	Oaxaca	Gulf	titia-SAtl
**MO0210**	M	titia	MO02	36.95	-90.99	2019-08-17	United States	Missouri	Gulf	titia-NAtl
**MO0211**	M	titia	MO02	36.95	-90.99	2019-08-17	United States	Missouri	Gulf	titia-NAtl
**MO0309**	M	titia	MO03	36.56	-90.35	2019-08-18	United States	Missouri	Gulf	titia-NAtl
**MO0311**	M	titia	MO03	36.56	-90.35	2019-08-18	United States	Missouri	Gulf	titia-NAtl
**MO0313**	M	titia	MO03	36.56	-90.35	2019-08-18	United States	Missouri	Gulf	titia-NAtl
**MO0351**	F	titia	MO03	36.56	-90.35	2019-08-18	United States	Missouri	Gulf	titia-NAtl

Sample Name	Sex	Species	Site ID	Lat	Long	Collection Date	Country	Province	Ocean drainage	Lineage assignment
**MS0108**	M	titia	MS01	32.52	-90.74	2019-08-21	United States	Mississippi	Gulf	titia-NAtl
**MS0114**	M	titia	MS01	32.52	-90.74	2019-08-21	United States	Mississippi	Gulf	titia-NAtl
**MS0116**	M	titia	MS01	32.52	-90.74	2019-08-21	United States	Mississippi	Gulf	titia-NAtl
**NA0103**	M	titia	NA01	9.74	-84.63	2014-05-13	Costa Rica		Pacific	titia-Pac
**NCRRb04**	M	titia	NCRR02	37.84	-78.81	2019-07-15	United States	Virginia	Atlantic	titia-NAtl
**NCRRb05**	M	titia	NCRR02	37.84	-78.81	2019-07-15	United States	Virginia	Atlantic	titia-NAtl
**NMSCa01**	M	titia	NMSC01	15.77	-93.3	2021-06-15	Mexico	Chiapas	Pacific	titia-Pac
**NMSCa02**	M	titia	NMSC01	15.77	-93.3	2021-06-15	Mexico	Chiapas	Pacific	titia-Pac
**NMSCa05**	M	titia	NMSC01	15.77	-93.3	2021-06-15	Mexico	Chiapas	Pacific	titia-Pac
**NPKBa05**	F	titia	NPKB1	31.52	-93.14	2017-07-10	United States	Louisiana	Gulf	titia-NAtl
**NPKBa06**	F	titia	NPKB1	31.52	-93.14	2017-07-10	United States	Louisiana	Gulf	titia-NAtl
**NPKBa10**	M	titia	NPKB1	31.52	-93.14	2017-07-10	United States	Louisiana	Gulf	titia-NAtl
**NR01.01**	M	titia	NR01	30.36	-94.09	2012-06-19	United States	Texas	Gulf	titia-NAtl
**NR02.06**	M	titia	NR02	30.68	-94.09	2012-06-19	United States	Texas	Gulf	titia-NAtl
**OK0101**	M	titia	OK01	35.24	-98.55	2019-08-31	United States	Oklahoma	Gulf	titia-NAtl
**PAa01**	M	titia	PA	18.55	-95.07	2011-04-09	Mexico	Veracruz	Gulf	titia-SAtl
**PAa02**	M	titia	PA	18.55	-95.07	2011-03-29	Mexico	Veracruz	Gulf	titia-SAtl
**PAa07**	F	titia	PA	18.55	-95.07	2011	Mexico	Veracruz	Gulf	titia-SAtl
**PC01.024**	M	titia	PC01	31.08	-93.49	2012-06-16	United States	Louisiana	Gulf	titia-NAtl

Sample Name	Sex	Species	Site ID	Lat	Long	Collection Date	Country	Province	Ocean drainage	Lineage assignment
**PCa01**	M	titia	PC01	31.08	-93.49	2012-06-16	United States	Louisiana	Gulf	titia-NAtl
**PCCC1**	M	titia	PCCC1	30.99	-89.01	2017-07-08	United States	Mississippi	Gulf	titia-NAtl
**PSa03**	M	titia	ps01	9.54	-84.28	2014-05-14	Costa Rica	Puntarenas	Pacific	titia-Pac
**PSa04**	M	titia	ps01	9.54	-84.28	2014-05-14	Costa Rica	Puntarenas	Pacific	titia-Pac
**PSa06**	B	titia	ps01	9.54	-84.28	2014-05-14	Costa Rica	Puntarenas	Pacific	titia-Pac
**PUMAa04**	F	titia	PUMA01	15.77	-93.33	2021-06-16	Mexico	Chiapas	Pacific	titia-Pac
**PUMAa08**	M	titia	PUMA01	15.77	-93.33	2021-06-16	Mexico	Chiapas	Pacific	titia-Pac
**RCJRa02**	M	titia	RCJR01	37.52	-77.47	2019-07-12	United States	Virginia	Atlantic	titia-NAtl
**RCJRa04**	M	titia	RCJR01	37.52	-77.47	2019-07-12	United States	Virginia	Atlantic	titia-NAtl
**RCJRa05**	M	titia	RCJR01	37.52	-77.47	2019-07-12	United States	Virginia	Atlantic	titia-NAtl
**RCJRb02**	M	titia	RCJR02	37.55	-77.52	2019-07-12	United States	Virginia	Atlantic	titia-NAtl
**RCJRb04**	M	titia	RCJR02	37.55	-77.52	2019-07-12	United States	Virginia	Atlantic	titia-NAtl
**RLPEa05**	M	titia	RLPE01	16.52	-95.07	2021-06-20	Mexico	Oaxaca	Pacific	titia-Pac
**RLPEa06**	M	titia	RLPE01	16.52	-95.07	2021-06-20	Mexico	Oaxaca	Pacific	titia-Pac
**RLPEa08**	M	titia	RLPE01	16.52	-95.07	2021-06-21	Mexico	Oaxaca	Pacific	titia-Pac
**RLPEa09**	M	titia	RLPE01	16.52	-95.07	2021-06-21	Mexico	Oaxaca	Pacific	titia-Pac
**RLPEb02**	M	titia	RLPE02	16.56	-95.1	2021-06-21	Mexico	Oaxaca	Pacific	titia-Pac
**SCSCa01**	F	titia	SCSC01	28.72	-81.31	2019-07-11	United States	Florida	Atlantic	titia-NAtl
**SCSCa02**	F	titia	SCSC01	28.72	-81.31	2019-07-11	United States	Florida	Atlantic	titia-NAtl
**SCSCa03**	F	titia	SCSC01	28.72	-81.31	2019-07-11	United States	Florida	Atlantic	titia-NAtl

Sample Name	Sex	Species	Site ID	Lat	Long	Collection Date	Country	Province	Ocean drainage	Lineage assignment
**SJ01.21**	M	titia	SJ01_US	30.21	-95.4	2012-06-19	United States	Texas	Gulf	titia-NAtl
**SMAA06**	M	titia	SMAA2	10.04	-83.33	2022-03-31	Costa Rica	Limón	Caribbean	titia-SAtl
**SMAA07**	MM	titia	SMAA2	10.04	-83.33	2022-04-11	Costa Rica	Limón	Caribbean	titia-SAtl
**ST0102**	M	titia	ST01	9.38	-84.02	2014-05-14	Costa Rica	Puntarenas	Pacific	titia-Pac
**ST0103**	M	titia	ST01	9.38	-84.02	2014-05-14	Costa Rica	Puntarenas	Pacific	titia-Pac
**STDMa04**	M	titia	STDM01	15.82	-96.69	2021-06-10	Mexico	Oaxaca	Pacific	titia-Pac
**STDMa06**	M	titia	STDM01	15.82	-96.69	2021-06-10	Mexico	Oaxaca	Pacific	titia-Pac
**TN0101**	M	titia	TN01	35.03	-89.35	2019-08-22	United States	Tennessee	Gulf	titia-NAtl
**TN0103**	M	titia	TN01	35.03	-89.35	2019-08-22	United States	Tennessee	Gulf	titia-NAtl
**TN0105**	M	titia	TN01	35.03	-89.35	2019-08-22	United States	Tennessee	Gulf	titia-NAtl
**TN0411**	M	titia	TN04	36.55	-87.14	2019-08-23	United States	Tennessee	Gulf	titia-NAtl
**TN0423**	M	titia	TN04	36.55	-87.14	2019-08-23	United States	Tennessee	Gulf	titia-NAtl
**TN0425**	M	titia	TN04	36.55	-87.14	2019-08-23	United States	Tennessee	Gulf	titia-NAtl
**TN0427**	M	titia	TN04	36.55	-87.14	2019-08-23	United States	Tennessee	Gulf	titia-NAtl
**TULIa03**	M	titia	TULI01	15.89	-93.5	2021-06-15	Mexico	Chiapas	Pacific	titia-Pac
**TULIa08**	F	titia	TULI01	15.89	-93.5	2021-06-15	Mexico	Chiapas	Pacific	titia-Pac
**TULIa09**	F	titia	TULI01	15.89	-93.5	2021-06-15	Mexico	Chiapas	Pacific	titia-Pac
**TXRSa02**	M	titia	TXRS01	16.65	-93.16	2021-06-17	Mexico	Chiapas	Gulf	titia-SAtl
**TXRSa06**	M	titia	TXRS01	16.65	-93.16	2021-06-17	Mexico	Chiapas	Gulf	titia-SAtl
**TXRSb01**	M	titia	TXRS02	16.65	-93.16	2021-06-17	Mexico	Chiapas	Gulf	titia-SAtl
**TXRSb03**	M	titia	TXRS02	16.65	-93.16	2021-06-17	Mexico	Chiapas	Gulf	titia-SAtl
**TXRSb05**	M	titia	TXRS02	16.65	-93.16	2021-06-17	Mexico	Chiapas	Gulf	titia-SAtl

Sample Name	Sex	Species	Site ID	Lat	Long	Collection Date	Country	Province	Ocean drainage	Lineage assignment
**TXRSb07**	M	titia	TXRS02	16.65	-93.16	2021-06-17	Mexico	Chiapas	Gulf	titia-SAtl
**WCKCa01**	M	titia	WCKC1	31.05	-90.17	2017-07-07	United States	Mississippi	Gulf	titia-NAtl
**WCKCa02**	M	titia	WCKC1	31.05	-90.17	2017-07-07	United States	Mississippi	Gulf	titia-NAtl
**WCKCa03**	M	titia	WCKC1	31.05	-90.17	2017-07-07	United States	Mississippi	Gulf	titia-NAtl
**ZANAa04**	M	titia	ZANA01	16.45	-94.34	2021-06-13	Mexico	Oaxaca	Pacific	titia-Pac
**ZANAa06**	M	titia	ZANA01	16.45	-94.34	2021-06-13	Mexico	Oaxaca	Pacific	titia-Pac
**05LT11**	M	americana	05lt	20.73	-103.62	2005-07-02	Mexico	Jalisco	Pacific	americana-S
**05PU01**	M	americana	05pu	19.5	-104.67	2005-06-30	Mexico	Jalisco	Pacific	calverti
**05SI11**	F	americana	05si	19.56	-104.79	2005-07-15	Mexico	Jalisco	Pacific	americana-S
**05SI24**	F	americana	05si	19.56	-104.79	2005-07-15	Mexico	Jalisco	Pacific	calverti
**10AP36**	f	americana	AP	19.45609	-96.4793	2010-08-26	Mexico	Veracruz		calverti
**EAFB014142**	M	americana	EAFB01	30.54	-86.86	2021-08-24	United States	Florida	Gulf	americana-N
**EAFB014144**	M	americana	EAFB01	30.54	-86.86	2021-08-24	United States	Florida	Gulf	americana-N
**EAFB014146**	M	americana	EAFB01	30.54	-86.86	2021-08-24	United States	Florida	Gulf	americana-N
**EAFB014149**	F	americana	EAFB01	30.54	-86.86	2021-08-24	United States	Florida	Gulf	americana-N
**EC0101**	m	americana	EC01	23.1403	-99.1158	2004-07-21	Mexico	Tamaulipas		americana-S
**GL23892**	M	americana	GL2	30.07	-99.24	2021-08-15	United States	Texas	Gulf	americana-N
**GL23898**	F	americana	GL2	30.07	-99.24	2021-08-15	United States	Texas	Gulf	americana-N
**HXRCaAM01**	M	americana	HXRC01	15.84	-96.33	2021-06-07	Mexico	Oaxaca	Pacific	calverti

Sample Name	Sex	Species	Site ID	Lat	Long	Collection Date	Country	Province	Ocean drainage	Lineage assignment
**HXRCcAM01**	M	americana	HXRC03	15.86	-96.31	2021-06-09	Mexico	Oaxaca	Pacific	americana-S
**HXRCcAM02**	F	americana	HXRC03	15.86	-96.31	2021-06-09	Mexico	Oaxaca	Pacific	americana-S
**HXRCcAM03**	F	americana	HXRC03	15.86	-96.31	2021-06-09	Mexico	Oaxaca	Pacific	americana-S
**IA0101**	M	americana	IA01	41.29	-94.15	2019-08-07	United States	Iowa	Gulf	americana-N
**IA0103**	F	americana	IA01	41.29	-94.15	2019-08-07	United States	Iowa	Gulf	americana-N
**IA0506**	M	americana	IA05	42.3	-92.02	2019-08-10	United States	Iowa	Gulf	americana-N
**IA0607**	F	americana	IA05	42.3	-92.02	2019-08-10	United States	Iowa	Gulf	americana-N
**IA0608**	M	americana	IA05	42.3	-92.02	2019-08-10	United States	Iowa	Gulf	americana-N
**IL0203**	F	americana	IL02	41.64	-88.07	2019-08-13	United States	Illinois	Gulf	americana-N
**IL0204**	M	americana	IL02	41.64	-88.07	2019-08-13	United States	Illinois	Gulf	americana-N
**IL0301**	F	americana	IL03	42.46	-89.24	2019-08-13	United States	Illinois	Gulf	americana-N
**IL0302**	M	americana	IL03	42.46	-89.24	2019-08-13	United States	Illinois	Gulf	americana-N
**MEBA014006**	M	americana	MEBA01	29.72	-99.08	2021-08-16	United States	Texas	Gulf	americana-N
**MI0407**	M	americana	MI04	42.38	-83.92	2021-08-20	United States	Michigan	Atlantic	americana-N
**MI0409**	F	americana	MI04	42.38	-83.92	2021-08-20	United States	Michigan	Atlantic	americana-N
**MI0504**	M	americana	MI05	43.46	-84.08	2021-08-23	United States	Michigan	Atlantic	americana-N
**MI0505**	M	americana	MI05	43.46	-84.08	2021-08-23	United States	Michigan	Atlantic	americana-N

Sample Name	Sex	Species	Site ID	Lat	Long	Collection Date	Country	Province	Ocean drainage	Lineage assignment
**MO0110**	M	americana	MO01	37.44	-90.96	2019-08-16	United States	Missouri	Gulf	americana-N
**MO0111**	F	americana	MO01	37.44	-90.96	2019-08-16	United States	Missouri	Gulf	americana-N
**MO0112**	M	americana	MO01	37.44	-90.96	2019-08-16	United States	Missouri	Gulf	americana-N
**MO0213**	F	americana	MO02	36.95	-90.99	2019-08-17	United States	Missouri	Gulf	americana-N
**MO0214**	M	americana	MO02	36.95	-90.99	2019-08-17	United States	Missouri	Gulf	americana-N
**MS0222**	M	americana	MS01	32.52	-90.74	2019-08-21	United States	Mississippi	Gulf	americana-N
**NMSCaAM03**	F	americana	NMSC01	15.77	-93.3	2021-06-15	Mexico	Chiapas	Pacific	calverti
**OK0102**	F	americana	OK01	35.24	-98.55	2019-08-31	United States	Oklahoma	Gulf	americana-N
**OK0104**	F	americana	OK01	35.24	-98.55	2019-08-31	United States	Oklahoma	Gulf	americana-N
**OK0105**	M	americana	OK01	35.24	-98.55	2019-08-31	United States	Oklahoma	Gulf	americana-N
**RG0101**	m	americana	RG01	18.4116	-95.1719	2004-05-11	Mexico	Veracruz		calverti
**RG0103**	f	americana	RG01	18.4116	-95.1719	2004-05-11	Mexico	Veracruz		calverti
**SJ01.20**	M	americana	SJ01_US	30.21	-95.4	2012-06-19	United States	Texas	Gulf	americana-N
**STDMaAM01**	M	americana	STDM01	15.82	-96.69	2021-06-10	Mexico	Oaxaca	Pacific	americana-S
**STDMaAM02**	M	americana	STDM01	15.82	-96.69	2021-06-10	Mexico	Oaxaca	Pacific	americana-S
**TN0119**	M	americana	TN01	35.03	-89.35	2019-08-22	United States	Tennessee	Gulf	americana-N
**TN0120**	M	americana	TN01	35.03	-89.35	2019-08-22	United States	Tennessee	Gulf	americana-N

Sample Name	Sex	Species	Site ID	Lat	Long	Collection Date	Country	Province	Ocean drainage	Lineage assignment
**TN0413**	F	americana	TN04	36.55	-87.14	2019-08-23	United States	Tennessee	Gulf	americana-N
**TN0414**	M	americana	TN04	36.55	-87.14	2019-08-23	United States	Tennessee	Gulf	americana-N
**TULIaAM01**	M	americana	TULI01	15.89	-93.5	2021-06-15	Mexico	Chiapas	Pacific	calverti
**TULIaAM02**	F	americana	TULI01	15.89	-93.5	2021-06-15	Mexico	Chiapas	Pacific	calverti
**TXRSaAM01**	F	americana	TXRS01	16.65	-93.16	2021-06-17	Mexico	Chiapas	Gulf	americana-S
**TXRSaAM04**	M	americana	TXRS01	16.65	-93.16	2021-06-17	Mexico	Chiapas	Gulf	americana-S
**TXRSbAM02**	M	americana	TXRS02	16.65	-93.16	2021-06-17	Mexico	Chiapas	Gulf	americana-S
**TXRSbAM03**	M	americana	TXRS02	16.65	-93.16	2021-06-17	Mexico	Chiapas	Gulf	americana-S
**TXRSbAM04**	F	americana	TXRS02	16.65	-93.16	2021-06-17	Mexico	Chiapas	Gulf	calverti
**WCKC14173**	M	americana	WCKC1	31.05	-90.17	2017-07-07	United States	Mississippi	Gulf	americana-N
**WCKC14176**	M	americana	WCKC1	31.05	-90.17	2017-07-07	United States	Mississippi	Gulf	americana-N

Supplementary Table 4: The number of votes received for each demographic scenario from the random forest classifier when applied to the observed data. For schematic diagrams of each demographic scenario see Supplementary Figure 2.

**Observed data**		**Model votes**														
**Species**	**Draft genome**	**1**	**2**	**3**	**4**	**5**	**6**	**7**	**8**	**9**	**10**	**11**	**12**	**13**	**Selected model**	**Post probability**
H. titia	*H. titia*	0	0	0	0	143	0	0	0	0	0	0	0	857	13	0.83
H. titia	*H. americana*	0	0	0	0	438	1	2	0	0	0	0	0	559	13	0.58
H. americana / calverti	*H. titia*	0	0	0	0	556	0	124	12	0	1	1	0	441	5	0.74
H. americana / calverti	*H. americana*	0	0	0	0	225	0	0	0	0	0	0	0	775	13	0.87

Supplementary Table 5: Summary statistics for each identified lineages of *Hetaerina titia* (South Atlantic, North Atlantic, Pacific) and across all lineages (all), excluding individuals from site CUAJ01/02 where the F1 individual was identified. Statistics calculated using the basic.stats, pi.dosage, and theta.Watt.dosage functions in the heirfstat (v0.5-11) package in R. Ho – observed heterozygosity, Hs – mean genetic diversity within each population, Ht – the total genetic diversity across all populations, Dst – the amount of genetic diversity among samples. Theta – the number of segregating sites within a population, pi - the between-sample nucleotide diversity

Population	Ho	Hs	Ht	Dst	theta	pi
South Atl	0.0961	0.1069	-	-	0.127	0.107
North Atl	0.0452	0.0559	-	-	0.046	0.056
Pacific	0.0395	0.075	-	-	0.055	0.075
All	0.0603	0.0792	0.2142	0.135	0.152	0.189

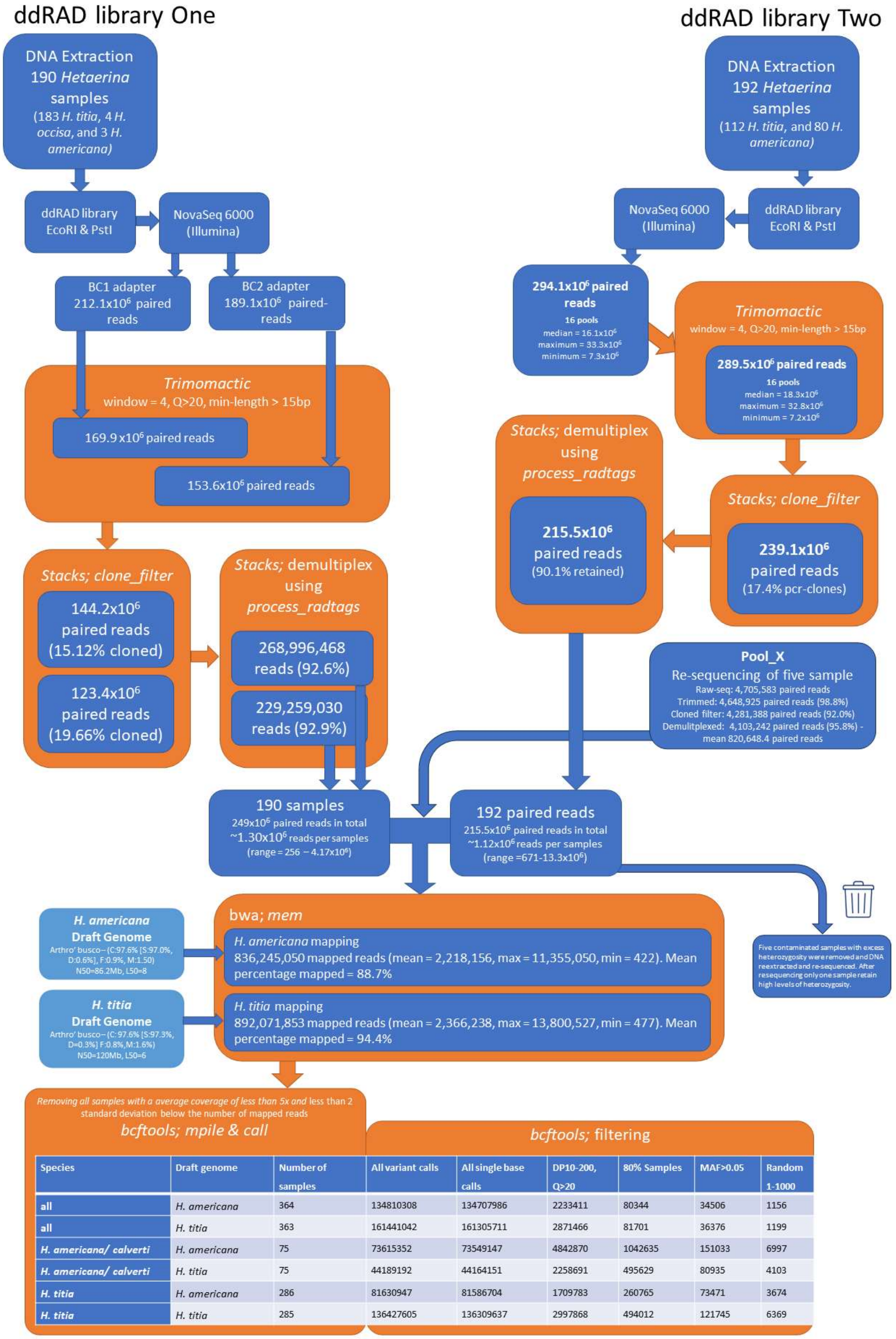

Removing all samples with a average coverage of less than 5x and less than 2 standard deviation below the number of mapped reads

bcftools; mpile & call | *bcftools;* filtering

Species	Draft genome	Number of samples	All variant calls	All single base calls	DP10-200, Q>20	80% Samples	MAF>0.05	Random 1-1000
**all**	*H. americana*	364	134810308	134707986	2233411	80344	34506	1156
**all**	*H. titia*	363	161441042	161305711	2871466	81701	36376	1199
H. americana/ calverti	*H. americana*	75	73615352	73549147	4842870	1042635	151033	6997
H. americana/ calverti	*H. titia*	75	44189192	44164151	2258691	495629	80935	4103
H. titia	*H. americana*	286	81630947	81586704	1709783	260765	73471	3674
H. titia	*H. titia*	285	136427605	136309637	2997868	494012	121745	6369

Supplementary Figure 1: Flow diagram of bioinformatics pipeline for ddRAD libraries. ddRAD one conducted at the NERC environmental omics facility (NEOF) using the ddRAD protocol from DaCosta and Sorenson (2014). Library Two was conducted at Durham University following the methodology from Franchini *et al.* (2017). For increased clarity, the final bottom table is also presented in Supplementary Table 1.

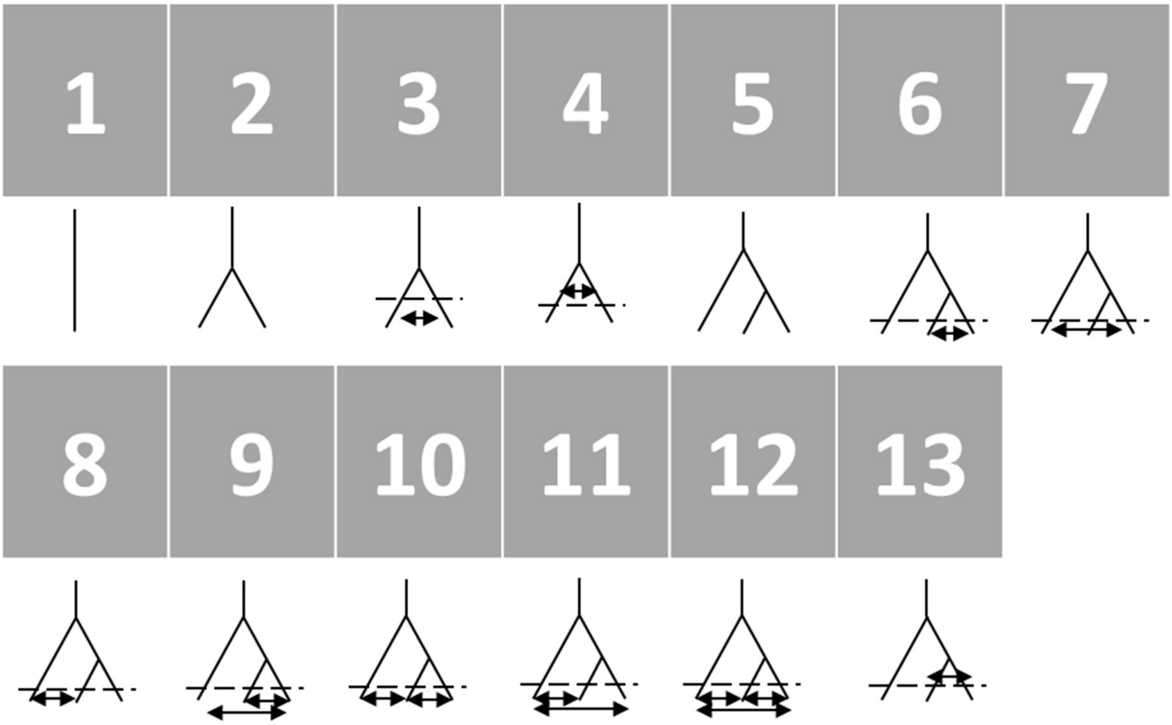

Supplementary Figure 2: Schematic diagrams of the thirteen demographic scenarios used in delimitR, We tested between all valid combination of the following demographic scenarios; one, two, or three potential lineages that arose either by pure isolation, isolation with gene flow, or isolation with contemporary geneflow, and one to three migration bands between lineages. Arrows represent which lineages migration was included between in each demographic scenario. The dashed lines indicate a distinction between migration that occurred during (isolation with gene flow) or after (contemporary geneflow) divergence, depending on whether the migration arrows are drawn above or below the dashed line respectively.

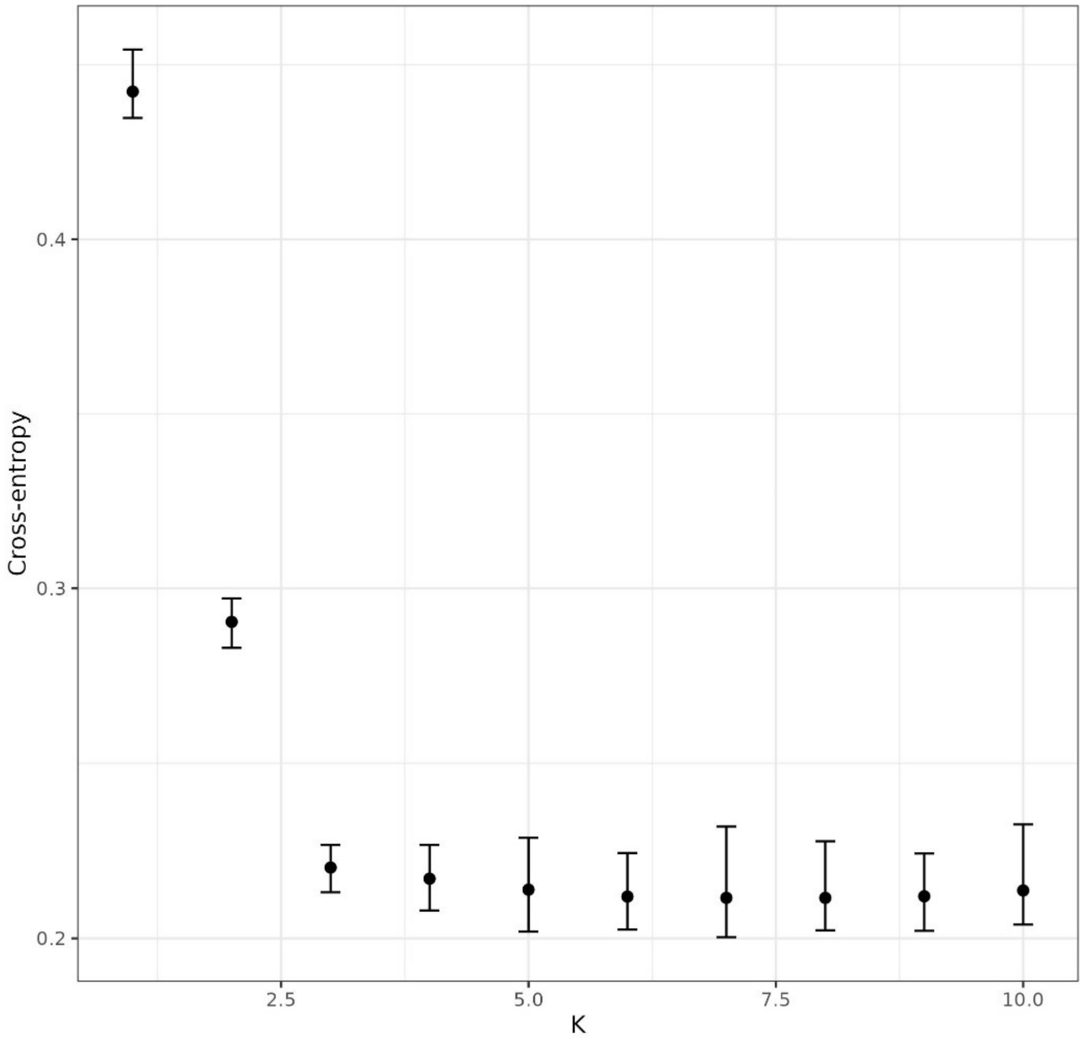

Supplementary Figure 3: The cross-entropy values from K = 1-10 and with error bars showing the maximum and minimum values from 100 repetitions of snmf analysis. Produced using the SNP library of *Hetaerina titia* samples mapped to the *H. titia* genome HetTit1.0 (Patterson et al., 2023)

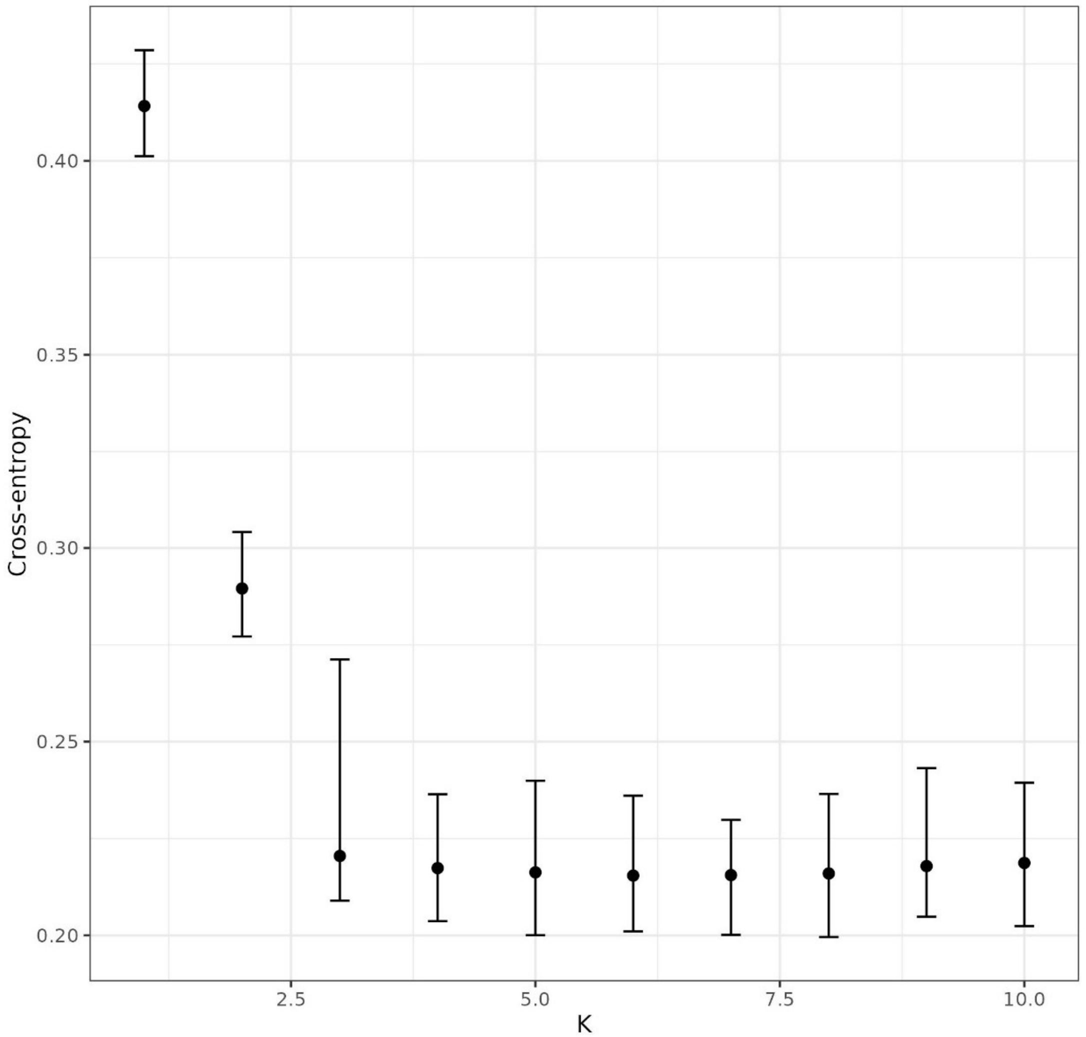

Supplementary Figure 4: The cross-entropy values from K = 1-10 and with error bars showing the maximum and minimum values from 100 repetitions of snmf analysis. Produced using the SNP library of *Hetaerina titia* samples mapped to the *H. americana* genome HetAmer1.0 (Grether et al., 2023)

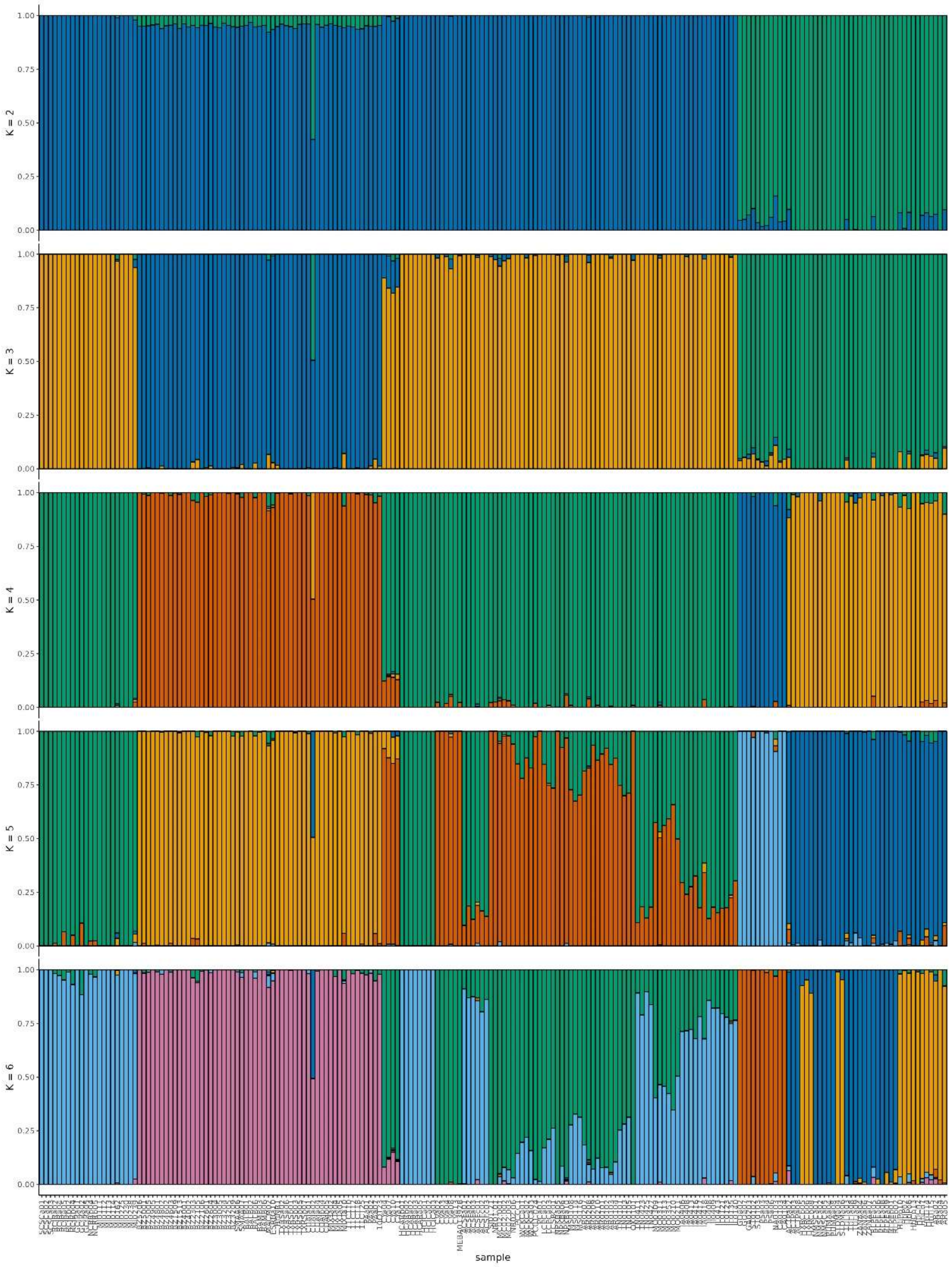

Supplementary Figure 5: Estimate of ancestry analysis for each individual using sNMF using K = 2-6 for all *Hetaerina titia* samples using the *Hetaerina titia* draft genome HetTit1.0 from (Patterson et al., 2023) Each value of K was run for 100 times with an alpha value of 100. Samples are ordered by drainage, then country, and then latitude.

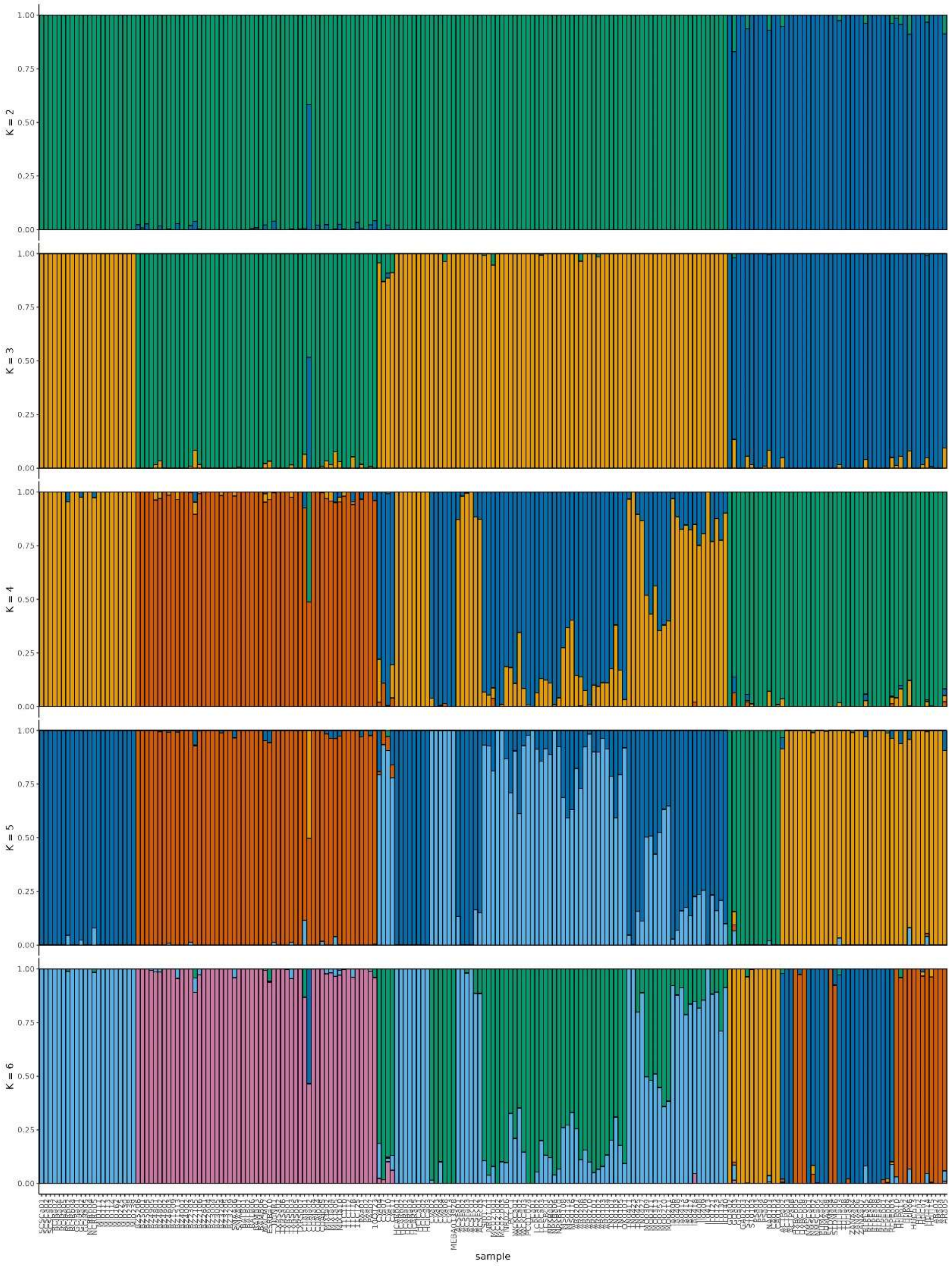

Supplementary Figure 6 :Estimate of ancestry analysis for each individual using sNMF using K = 2-6 for all *Hetaerina titia* samples using the *Hetaerina americana* draft genome HetAmer1.0 from (Grether et al., 2023) Each value of K was run for 100 times with an alpha value of 100.

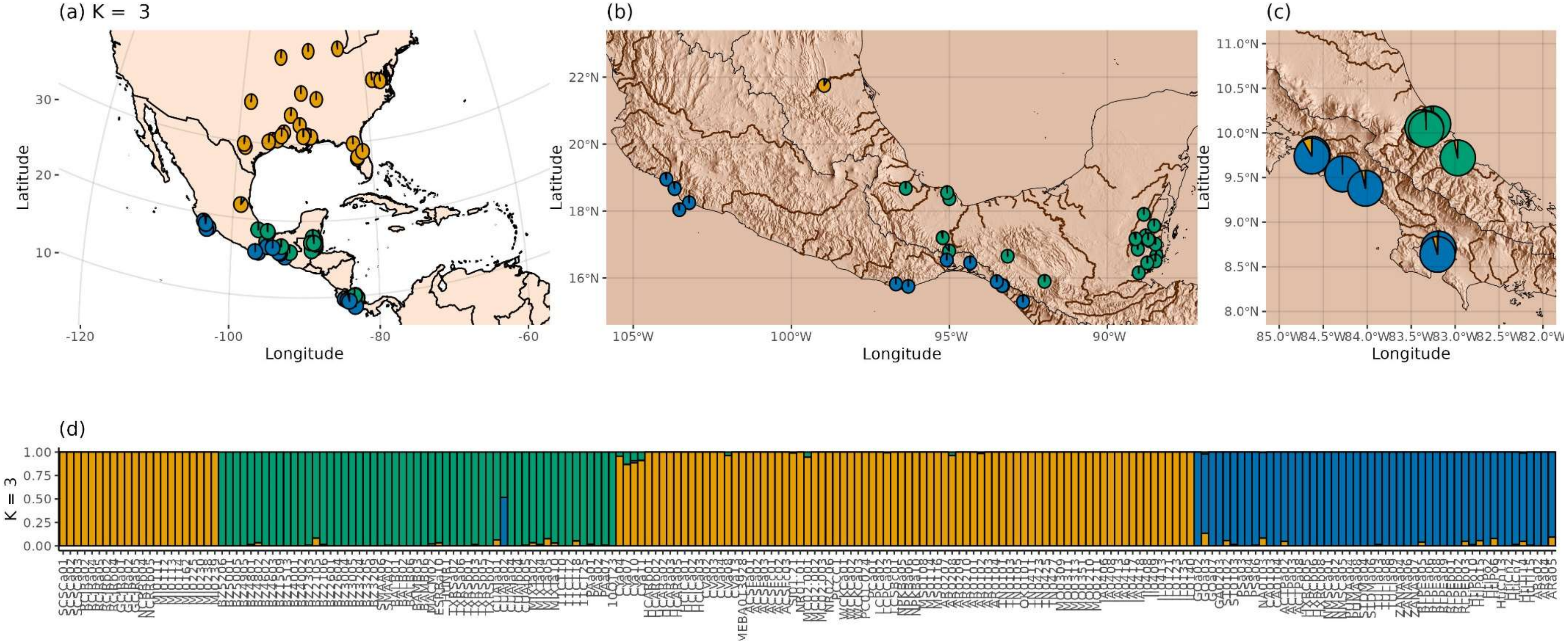

Supplementary Figure 7: Ancestry estimates using three ancestral populations for *Hetaerina titia*. SNPs were generated by mapping ddRAD reads to the draft genome of *H. americana.* This is in comparison to the Figure 1 in the main text which used the reads mapped to the draft genome of *H. titia*. LEA was run for 20 repetitions and an alpha value of 100. (a) The mean estimate of ancestry proportion for all samples within each sample site of *Hetaerina titia* across North and Central America, (b) Isthmus of Tehuantepec and Belize, and (c) Costa Rica. (d) Estimate of ancestry analysis for each individual. Samples are ordered by drainage, then country, and then latitude. Rivers and drainage basins from Hydrosheds. Topography data from the R package elevatr.

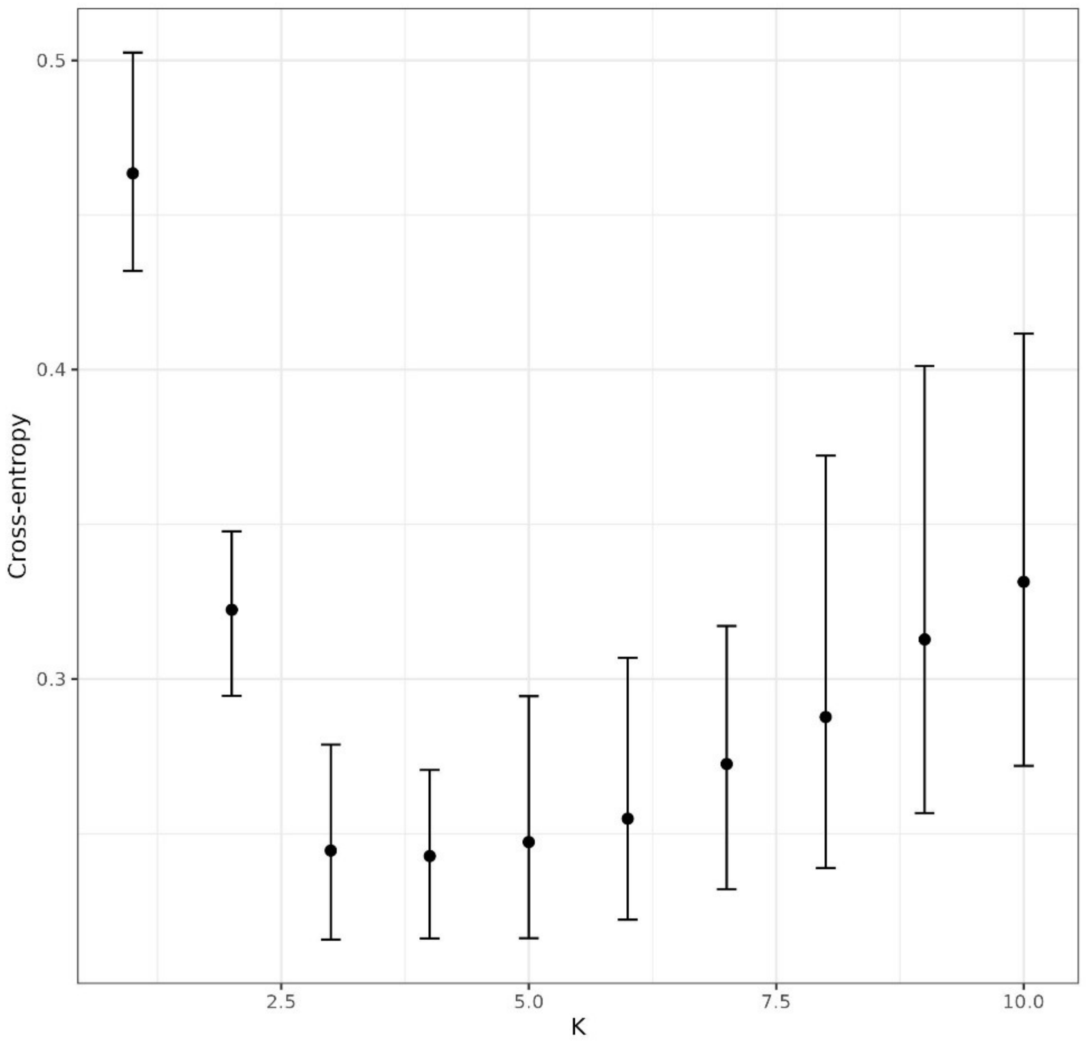

Supplementary Figure 8: The cross-entropy values from K = 1-10 and with error bars showing the maximum and minimum values from 100 repetitions of snmf analysis. Produced using the SNP library of *Hetaerina americana* sensu lato samples mapped to the *H. titia* genome HetTit1.0 (Patterson et al., 2023)

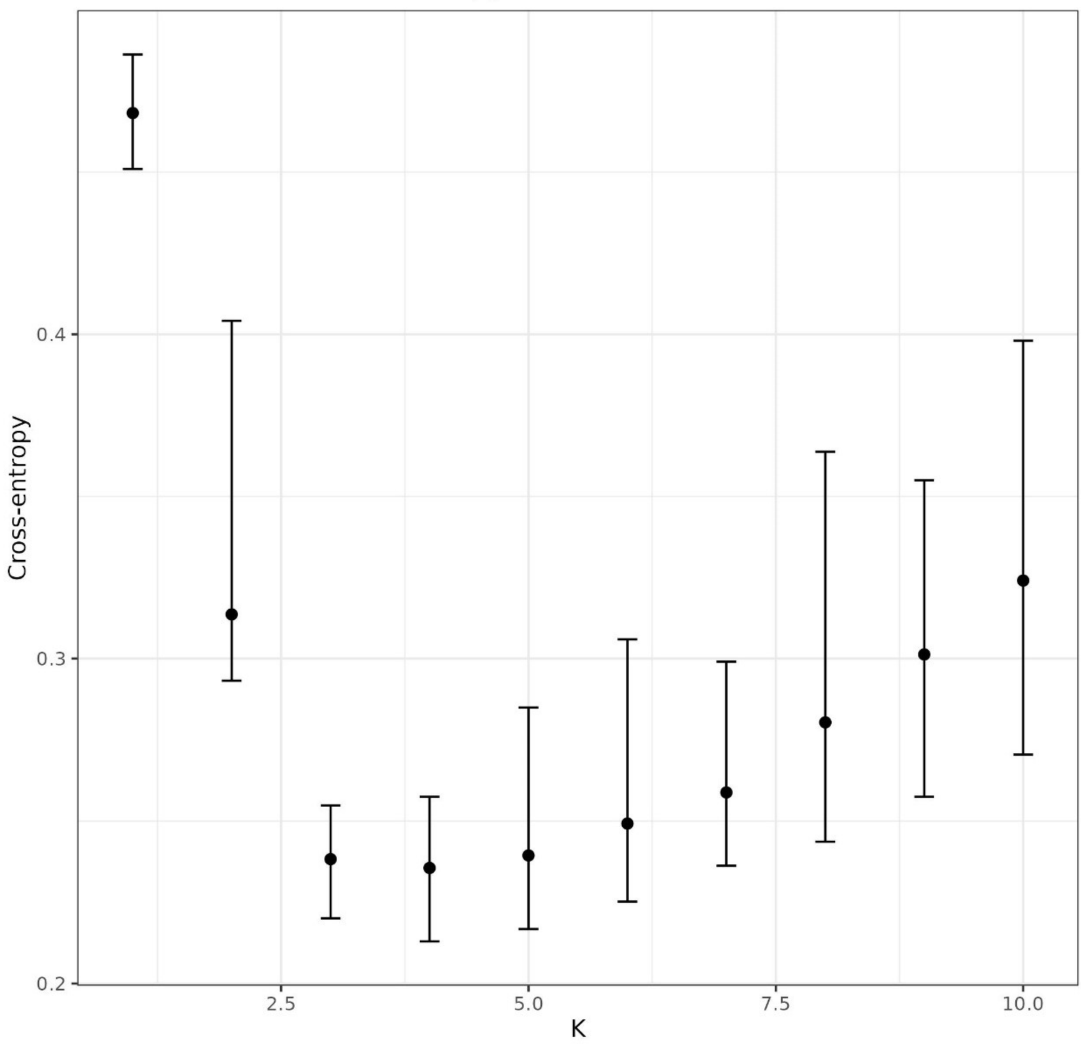

Supplementary Figure 9: The cross-entropy values from K = 1-10 and with error bars showing the maximum and minimum values from 100 repetitions of snmf analysis. Produced using the SNP library of *Hetaerina americana* sensu lato samples mapped to the *H. americana* genome HetAmer1.0 (Grether et al., 2023).

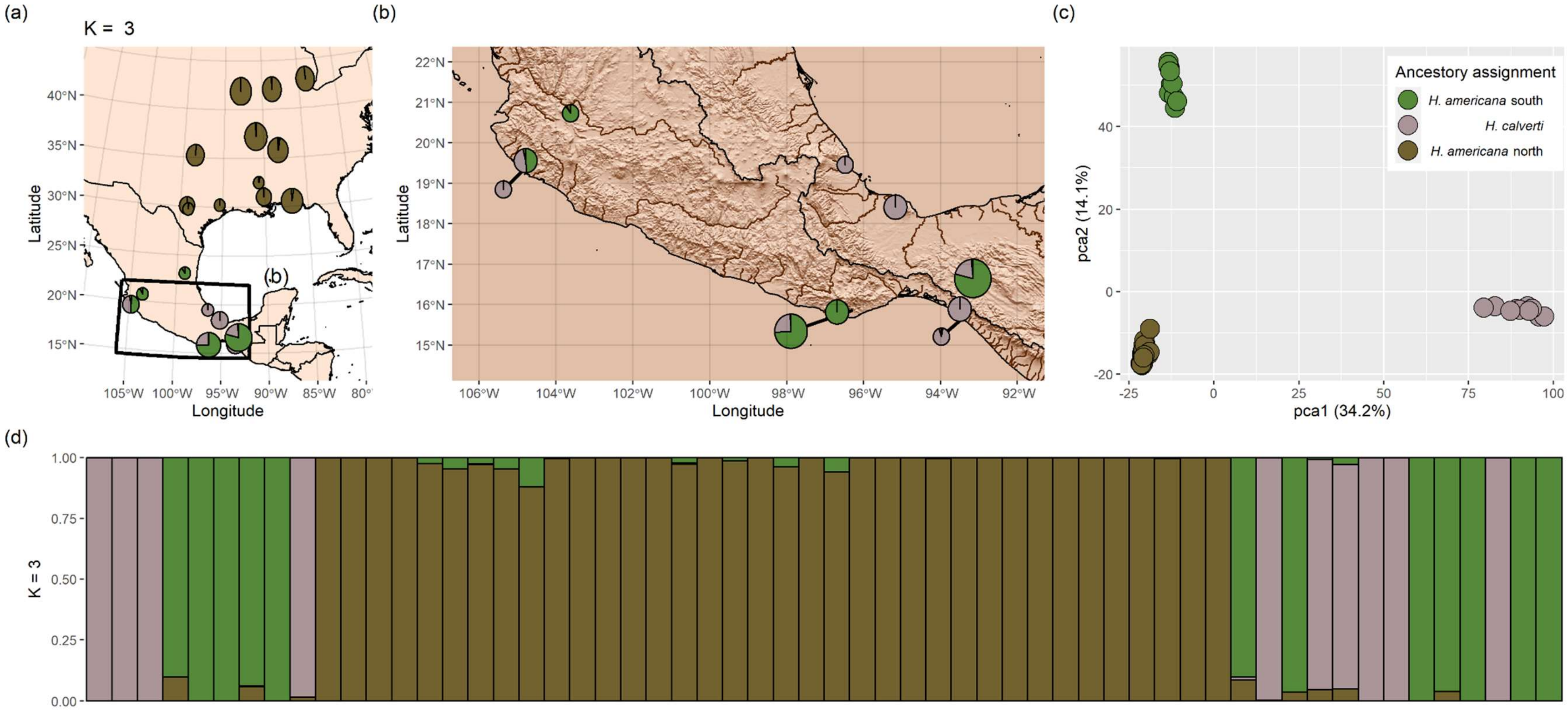

Supplementary Figure 10: Ancestry estimates using three ancestral populations for 58 *Hetaerina americana* (green and gold) and *H. calverti* (light grey) with a dataset of 5,259 unlinked biallelic autosomal SNPs, 20 repetitions and an alpha value of 100. SNPs where generated from reads mapped to the draft genome of *H. titia* **(a, b)** The mean estimate of ancestry proportion for all samples within each sample site of *H. americana/calverti*. **(c)** Principal component analysis of the same dataset with sample colour indicating the highest assigned ancestry population from sNMF for each individual. **(d)** Estimate of ancestry for each sample. Samples are ordered by drainage, then country, and then latitude. Rivers and drainage basins from Hydrosheds. Topography data downloaded using R package *elevatr*.

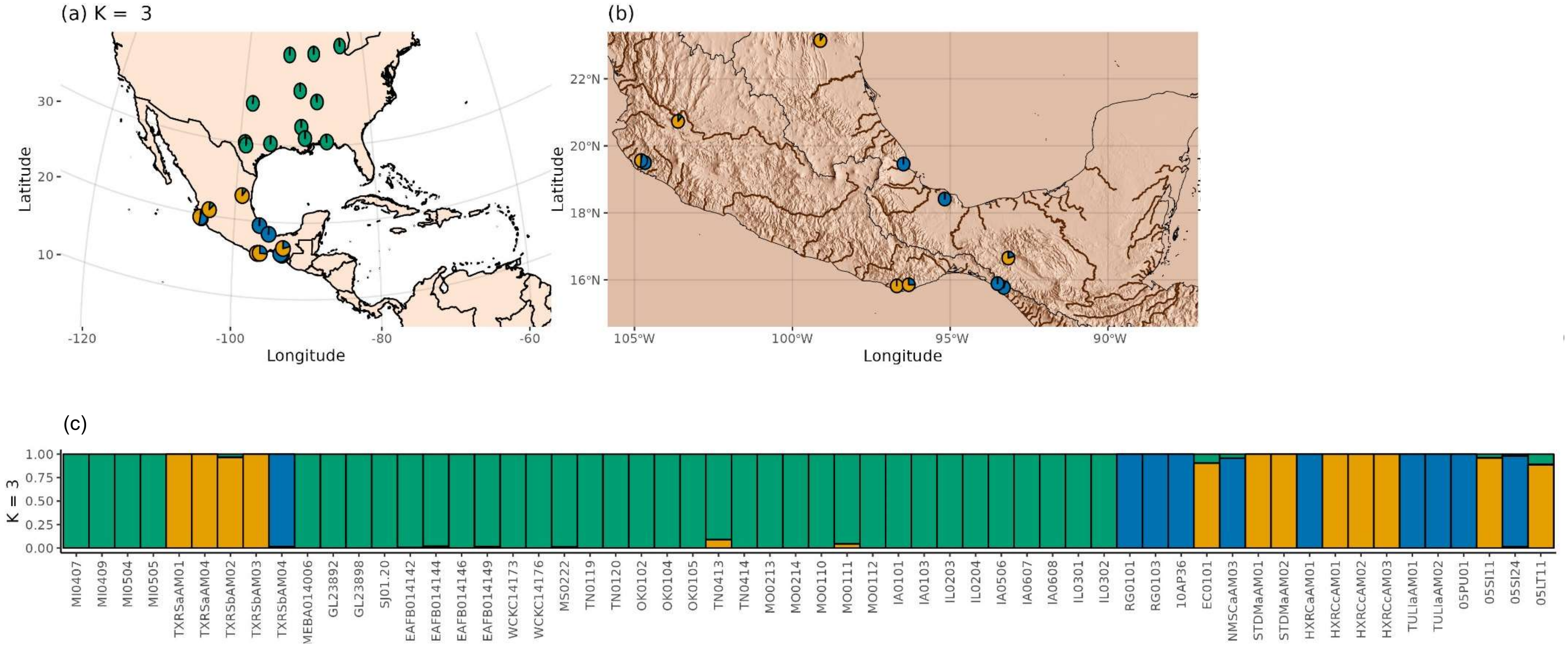

Supplementary Figure 11: Ancestry estimates using three ancestral populations for *Hetaerina americana* sensu lato. SNPs were generated by mapping ddRAD reads to the draft genome of *H. americana*. LEA was run for 20 repetitions and an alpha value of 100. (a) The mean estimate of ancestry proportion for all samples within each sample site of *Hetaerina americana* sensu lato across North and Central America, and (b) Isthmus of Tehuantepec and Belize. (c) Estimate of ancestry analysis for each individual. Samples are ordered by drainage, then country, and then latitude. Rivers and drainage basins from Hydrosheds. Topography data from the R package elevatr

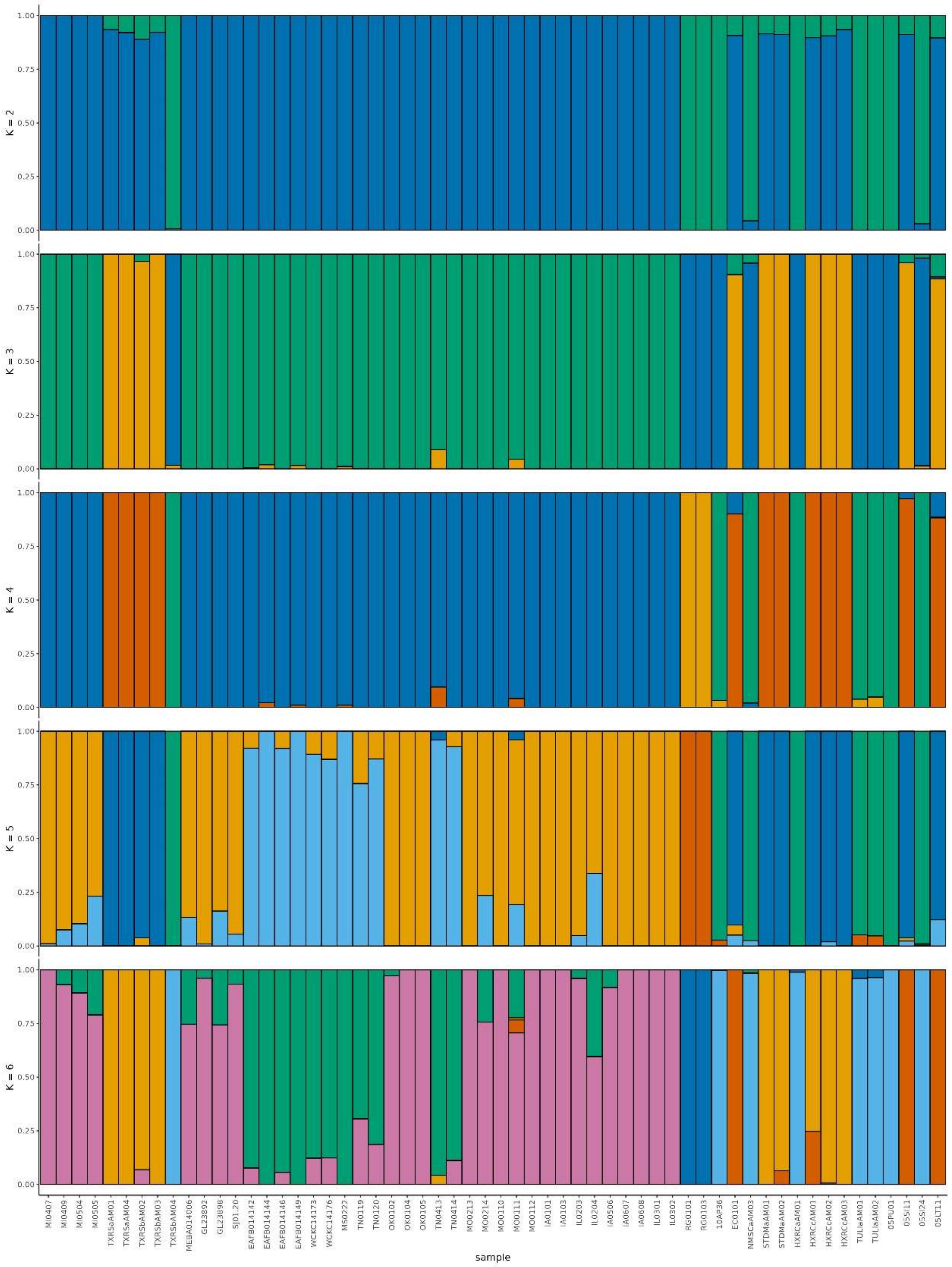

Supplementary Figure 12: Estimate of ancestry analysis using sNMF using K = 2-6 for all *Hetaerina americana* sensu lato samples using the *Hetaerina titia* draft genome HetTit1.0 from (Patterson et al., 2023). Each value of K was run for 100 times with an alpha value of 100.

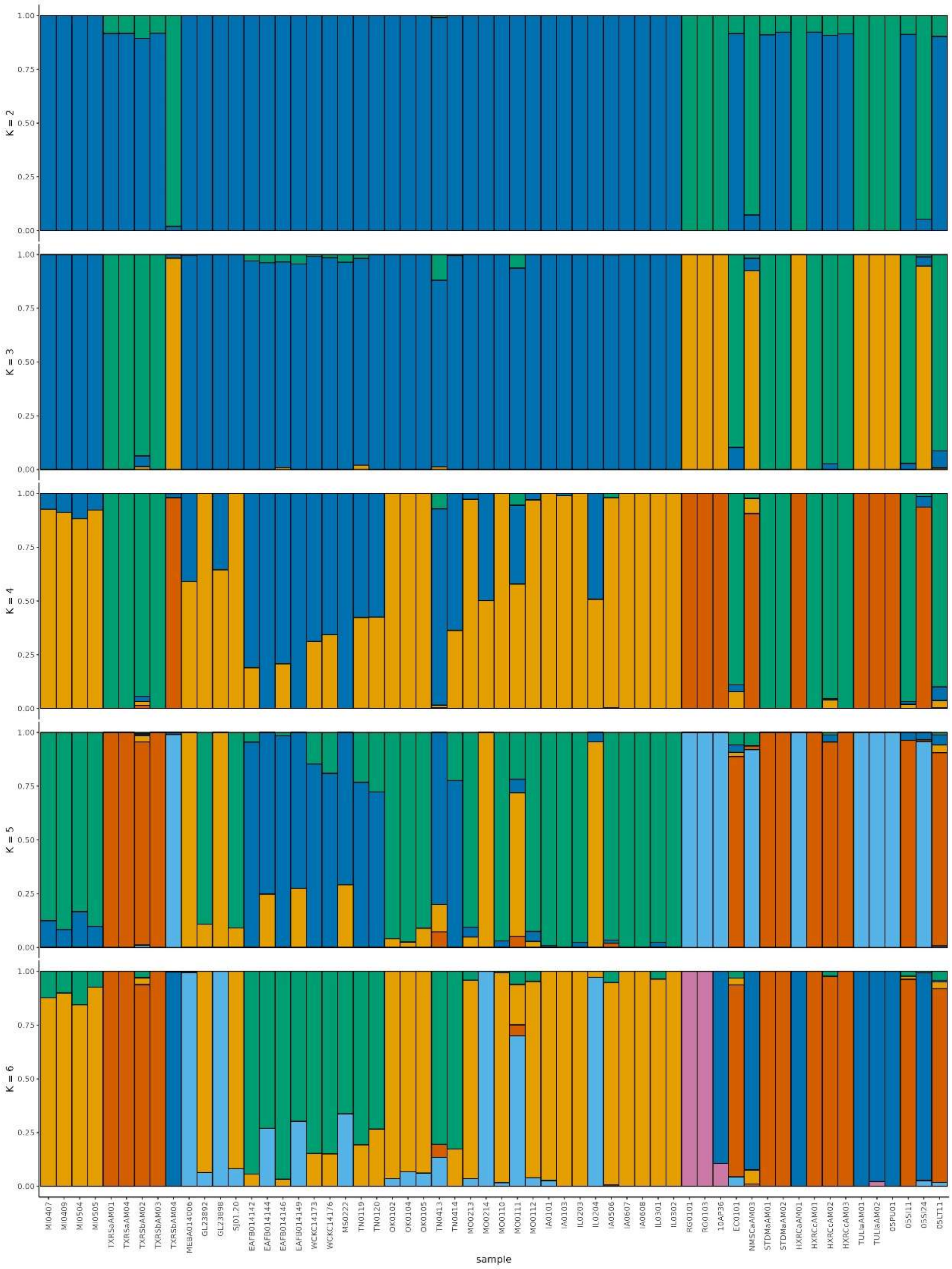

Supplementary Figure 13: Estimate of ancestry analysis using sNMF using K = 2-6 for all *Hetaerina americana* sensu lato samples using the *Hetaerina americana* draft genome HetAmer1.0 from (Grether et al., 2023) Each value of K was run for 100 times with an alpha value of 100.

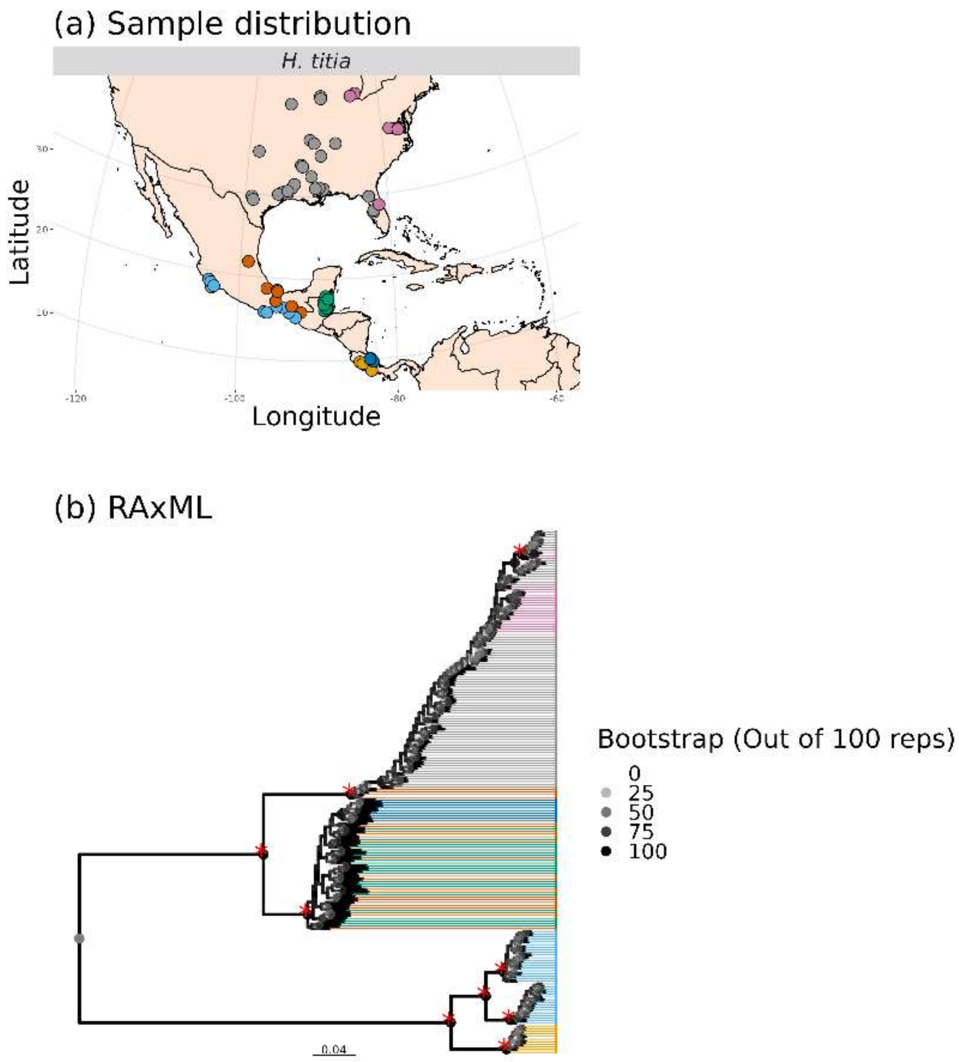

Supplementary Figure 14: The maximum likelihood tree for *Hetaerina titia* calculated by RAxML using a SNP library mapped onto the genome of *H. titia*. The tree is rooted around the midpoint which corresponds the root identified by the multi-species tree. The nodes are coloured by the number of bootstrap support values (out of 100). Nodes with greater than 95% support are marked with a red "*". The tree tips are coloured according to the species, the country, and river drainage of each sample shown in the inset map. Scale bar indicates number of substitutions per SNP site.

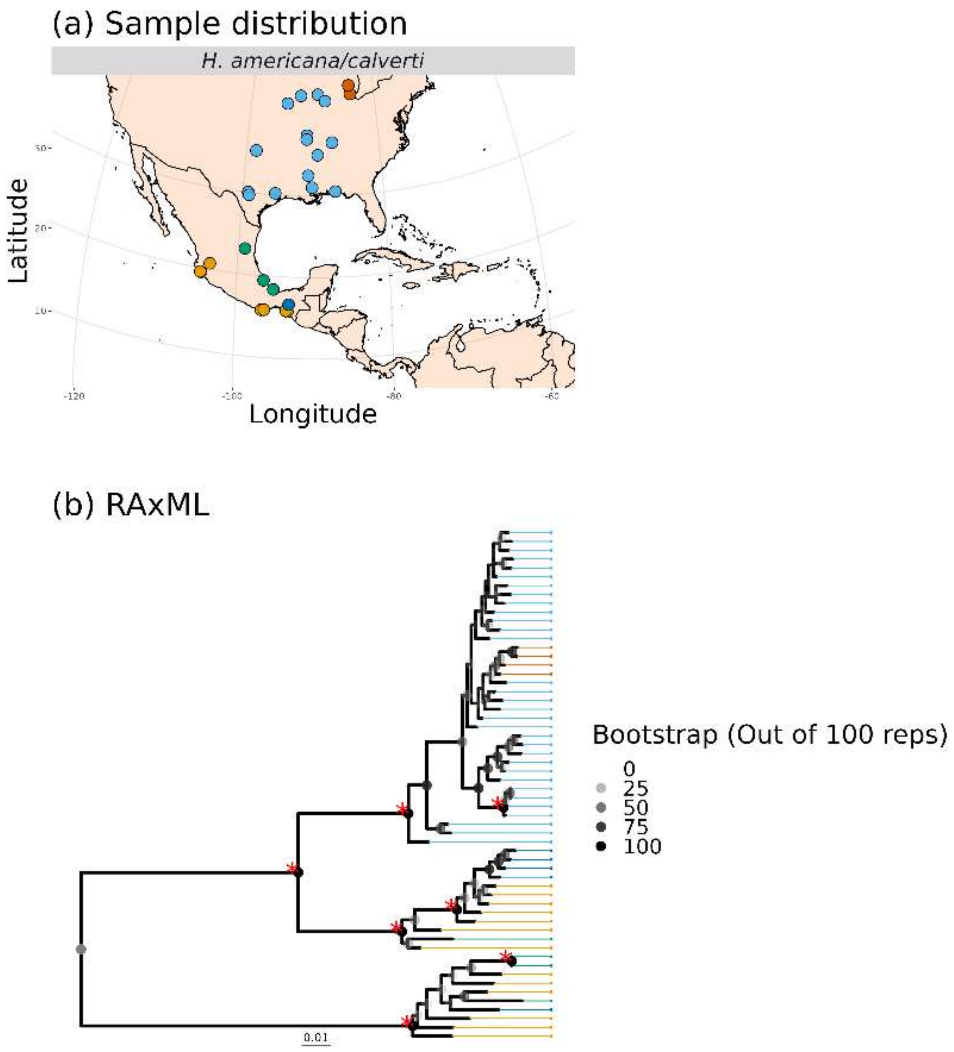

Supplementary Figure 15: The maximum likelihood tree for *Hetaerina americana and Hetaerina calverti* calculated by RAxML using a SNP library mapped onto the genome of *H. titia*. The tree is rooted around the midpoint which corresponds the root identified by the multi-species tree. The nodes are coloured by the number of bootstrap support values (out of 100). Nodes with greater than 95% support are marked with a red "*". The tree tips are coloured according to the species, the country, and river drainage of each sample shown in the inset map. The colour of each tip and geographical position of each sample does not separate *Hetaerina americana* and *Hetaerina calverti.* Scale bar indicates number of substitutions per SNP site.

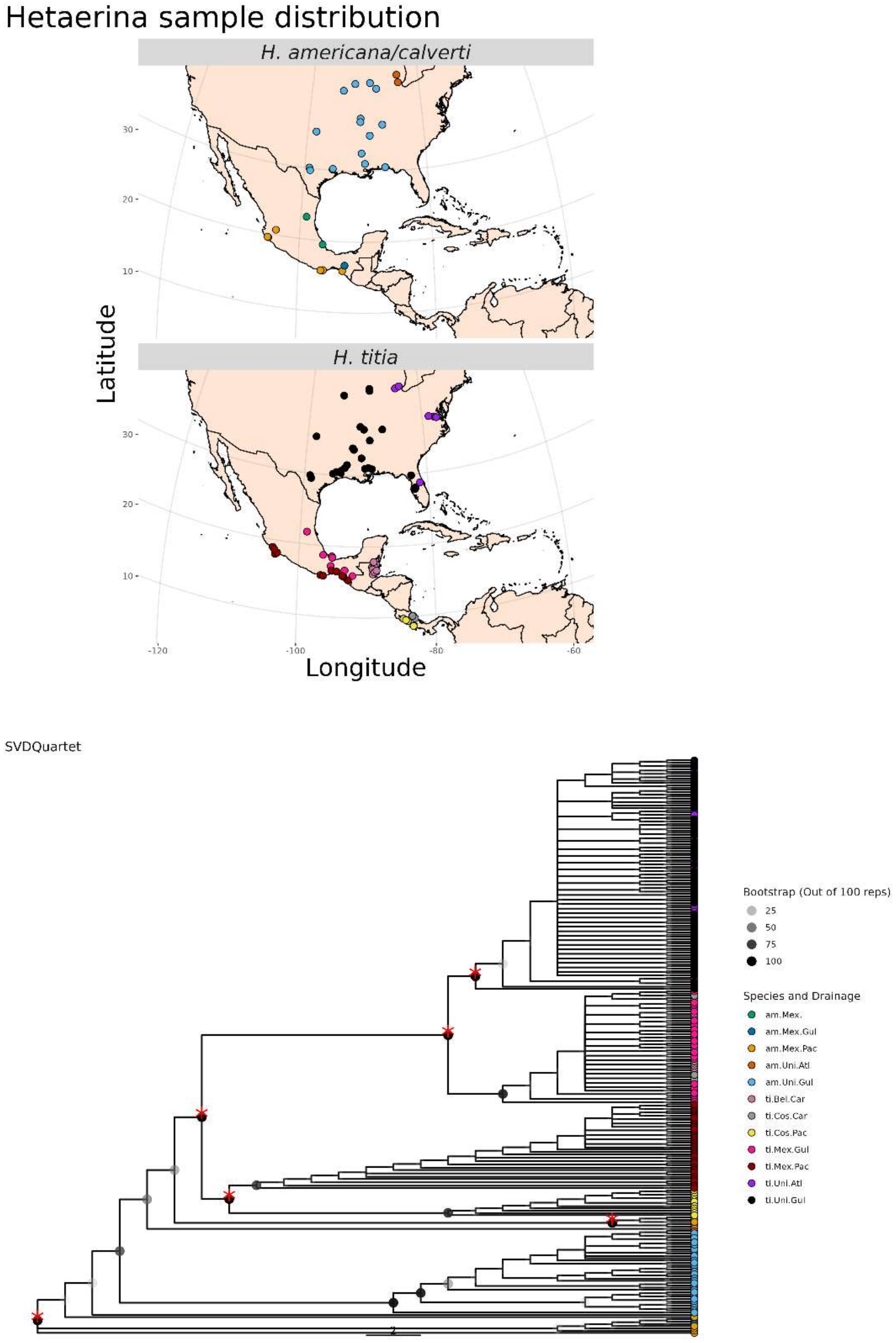

Supplementary Figure 16: SVDquartet analysis of *H. titia, H. americana* and *H. calverti* mapped to the draft genome of H. titia. Node with greater than 95% bootstrap support indicated with a read "*".

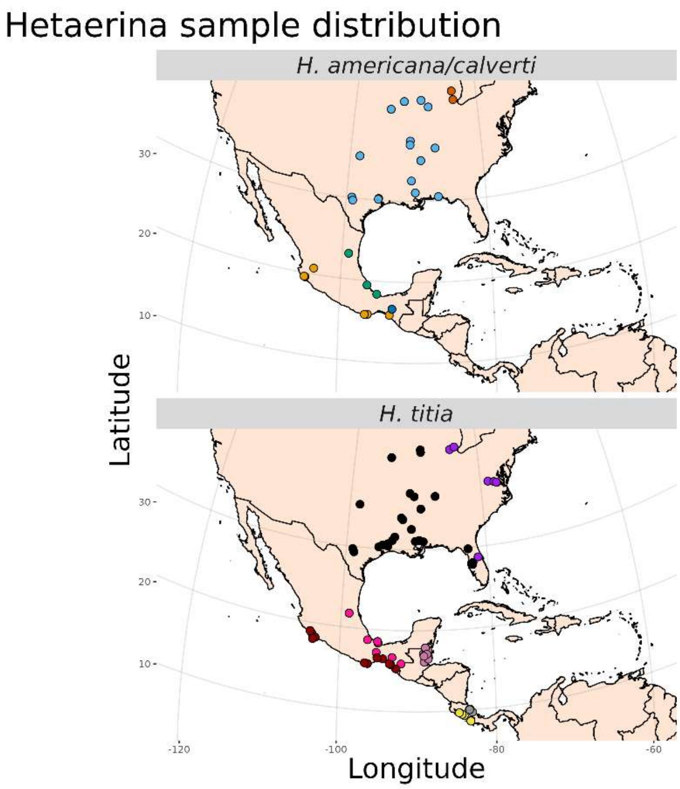

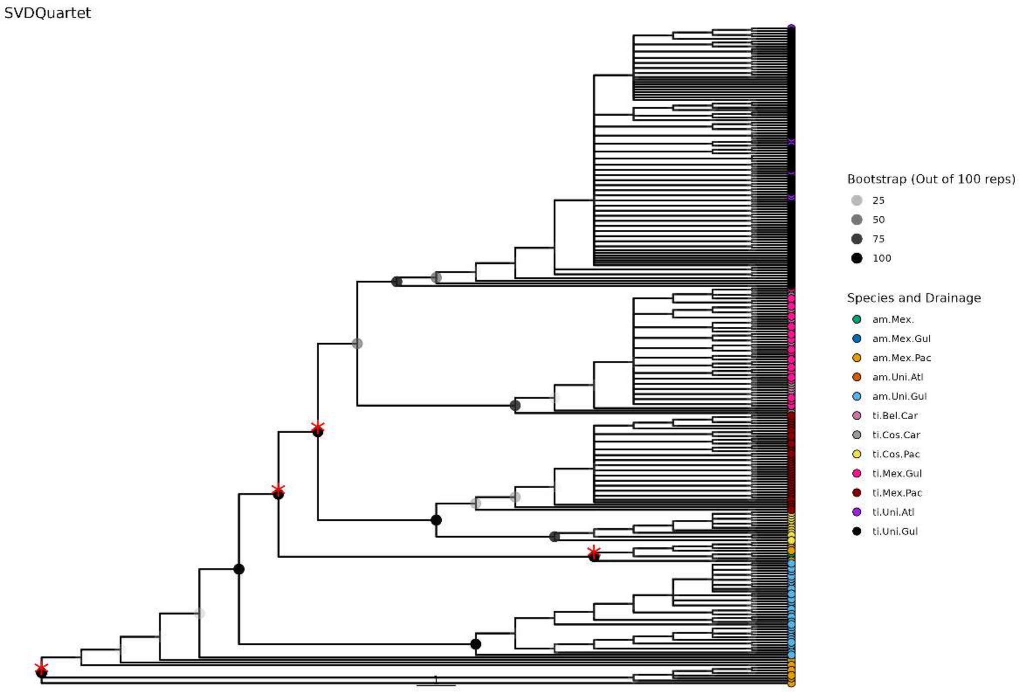

Supplementary Figure 17: SVDquartet analysis of *H. titia, H. americana* and *H. calverti* mapped to the draft genome of *H. americana.* Node with greater than 95% bootstrap support indicated with a read "*".

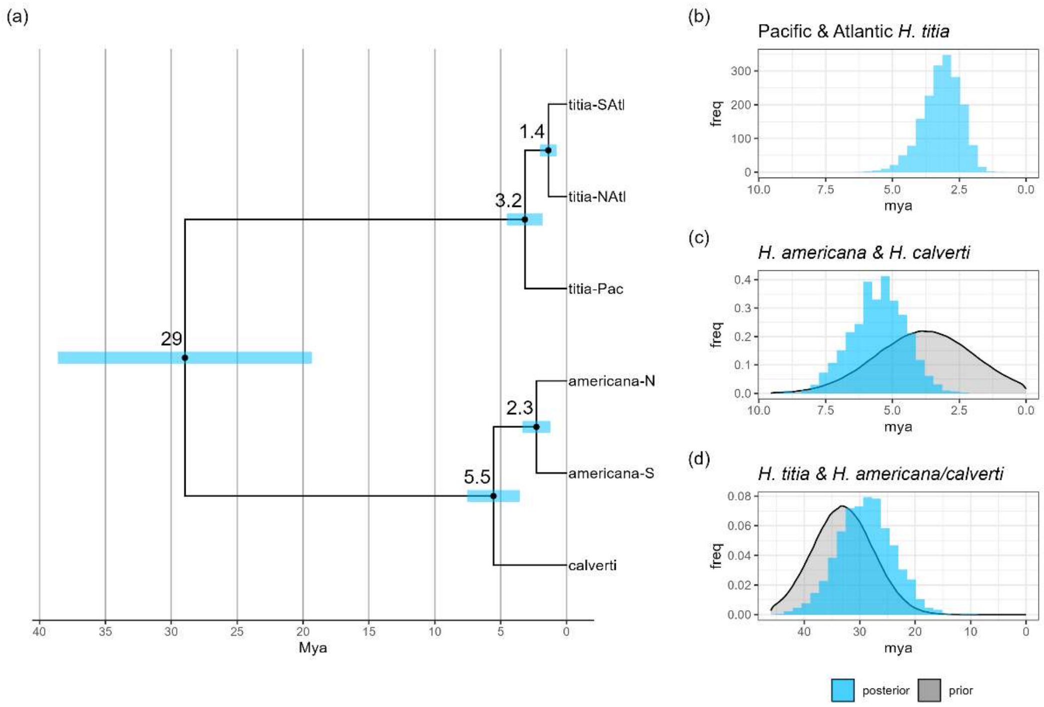

Supplementary Figure 18: Estimates of divergence dates between populations of *Hetaerina titia* (titia-NAtl, titia-SAlt, and titia-Pac), *Hetaerina americana* (americana-N americana-S), and *Hetaerina calverti* (calverti) calculated using SNAPP analysis in beast. Input data was 540 SNPs with 4 samples per tip, mapped to the genome of *Hetaerina americana*. Node labels indicate the mean estimated divergence date with 95% highest posterior density in blue. All branches had a posterior distribution of 1.

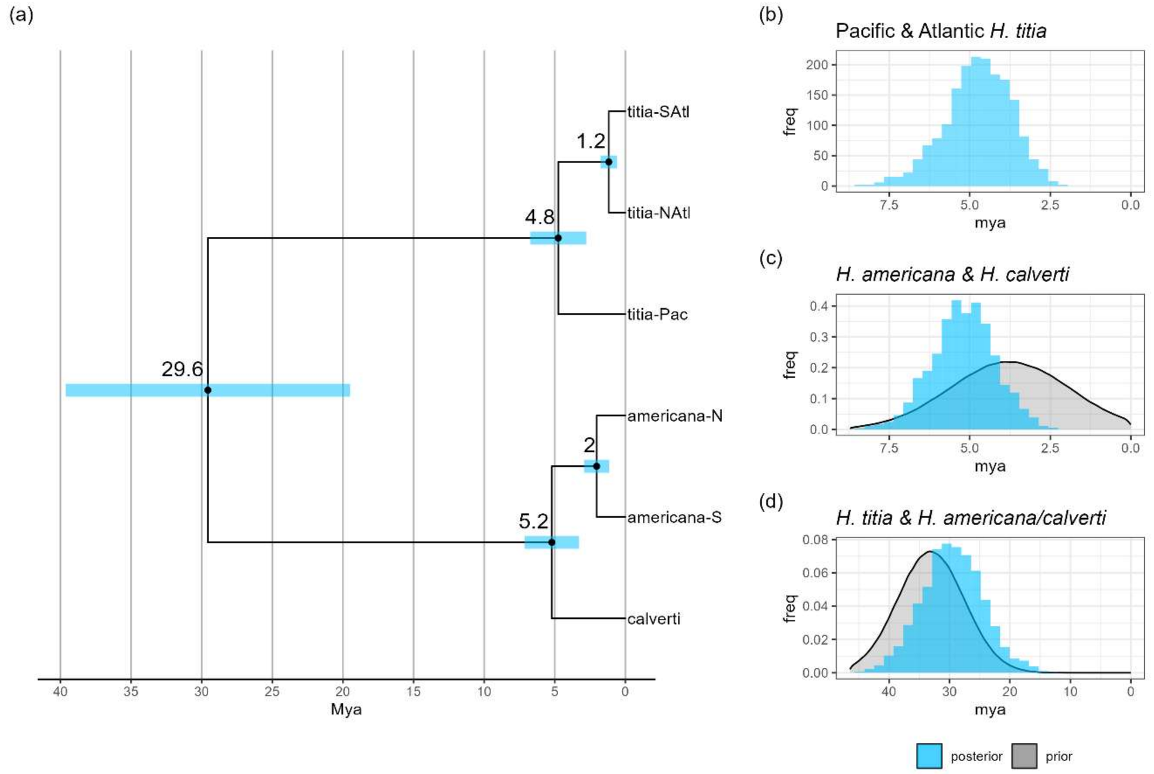

Supplementary Figure 19: Estimates of divergence dates between populations of *Hetaerina titia* (titia-NAtl, titia-SAlt, and titia-Pac), *Hetaerina americana* (americana-N americana-S), and *Hetaerina calverti* (calverti) calculated using SNAPP analysis in beast. Input data was 552 SNPs with four samples per tip, mapped to the genome of *Hetaerina titia*. Node labels indicate the mean estimated divergence date with 95% highest posterior density in blue. All branches had a posterior distribution of 1.

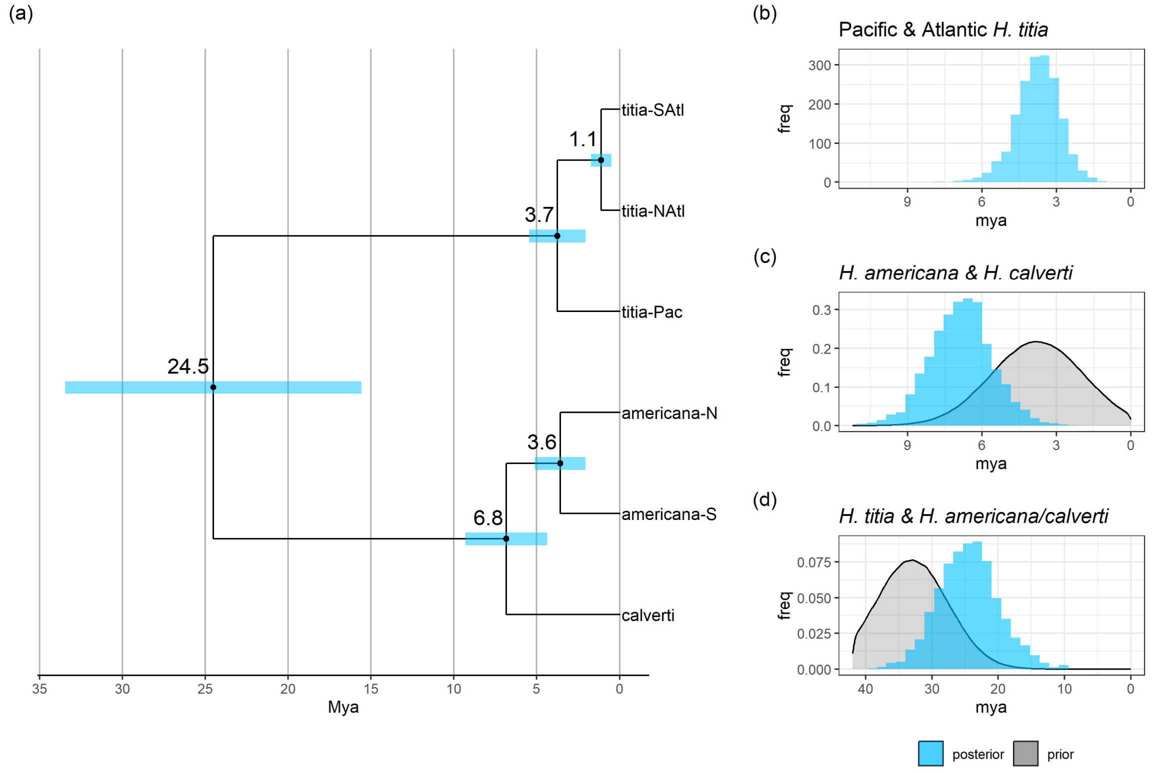

Supplementary Figure 20: Estimates of divergence dates between populations of *Hetaerina titia* (titia-NAtl, titia-SAlt, and titia-Pac), *Hetaerina americana* (americana-N americana-S), and *Hetaerina calverti* (calverti) calculated using SNAPP analysis in beast. Input data was 519 SNPs with three samples per tip, mapped *de novo* using ipyrad. Node labels indicate the mean estimated divergence date with 95% highest posterior density in blue. All branches had a posterior distribution of 1.

Conspecific Loci calling

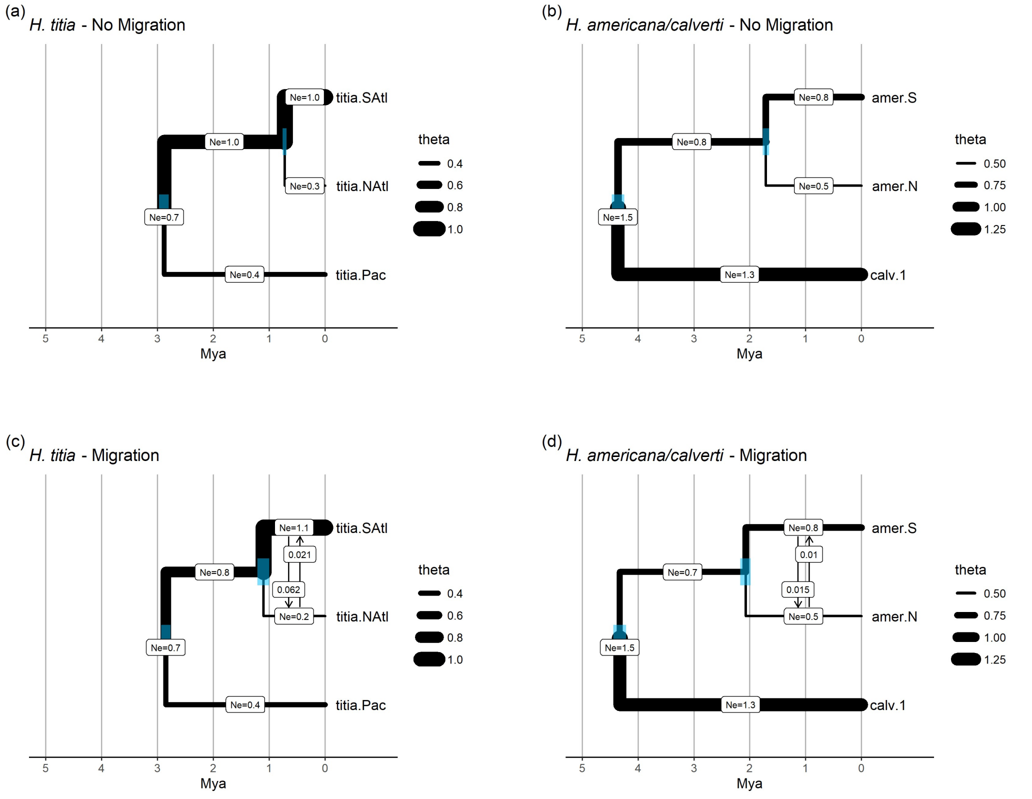

Supplementary Figure 21: The estimated divergence times (million years ago) and effective population size (theta – N*e* in millions of individuals) from G-PhoCS analysis of *H. titia* and *H. americana.* Migration rate is shown as the number of individuals per generation. All models ran for 1,000,000 interactions with 10% burn in. Blue bars show 95% highest posterior density for each divergence date **(a)** Model estimates for *H. titia* with no migration bands. **(b)** Model estimates for *H. americana* and *H. calverti* with no migration bands. **(c)** Model estimates for *H. titia* demography with migration bands between Northern and Southern Atlantic *H. titia* **(d)** Model estimates for *H. americana* and *H. calverti* with migration bands between North and Southern *H. americana*. G-PhoCS runs presented here are conducted on the conspecific draft genome. i.e., *H. titia* loci mapped to the *H. titia* draft genome and the *H. americana* loci mapped to the *H. americana* draft genome.

Heterospecific Loci calling

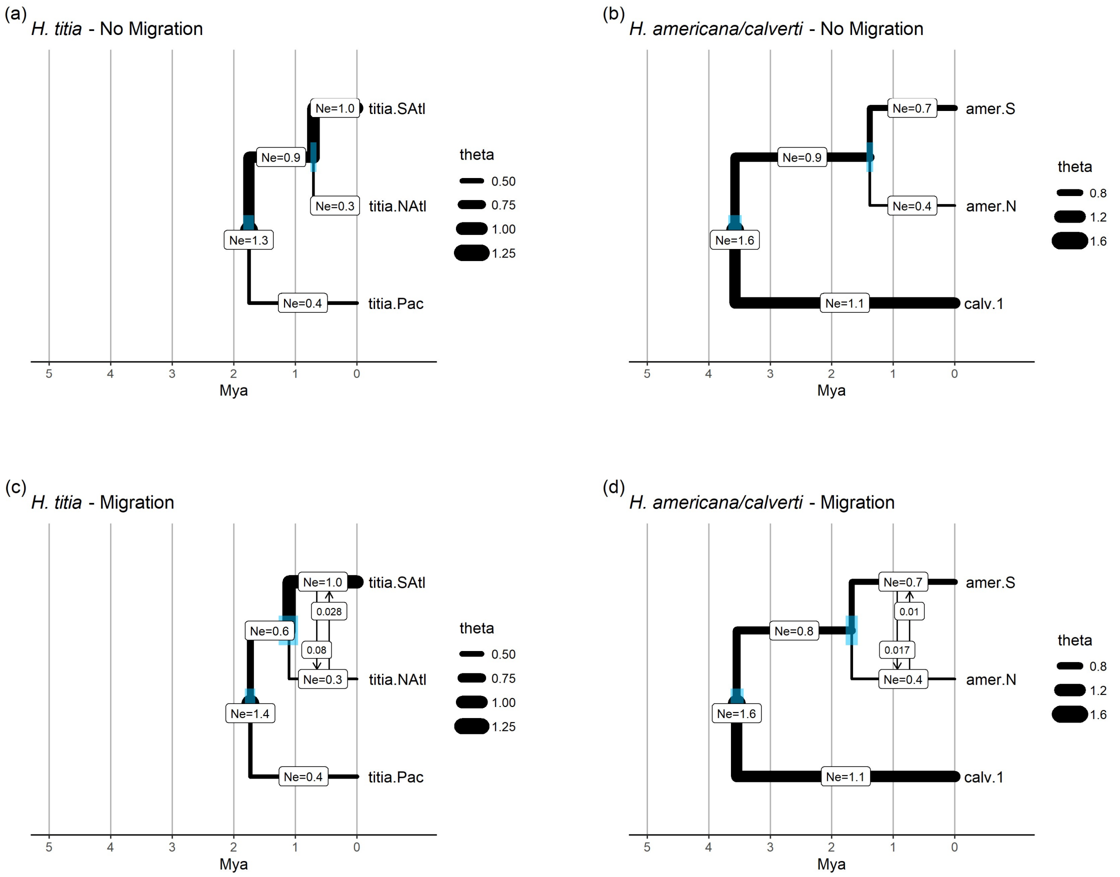

Supplementary Figure 22: The estimated divergence times (million years ago) and effective population size (theta – N*e* in millions of individuals) from G-PhoCS analysis of *H. titia* and *H. americana.* Migration rate is shown as the number of individuals per generation. All models ran for 1,000,000 interactions with 10% burn in. Blue bars show 95% highest posterior density for each divergence date **(a)** Model estimates for *H. titia* with no migration bands. **(b)** Model estimates for *H. americana* and *H. calverti* with no migration bands. **(c)** Model estimates for *H. titia* demography with migration bands between Northern and Southern Atlantic *H. titia* **(d)** Model estimates for *H. americana* and *H. calverti* with migration bands between North and Southern *H. americana*. G-PhoCS runs presented here are conducted on the heterospecific draft genome. i.e., *H. titia* loci mapped to the *H. americana* draft genome and the *H. americana* loci mapped to the *H. titia* draft genome.

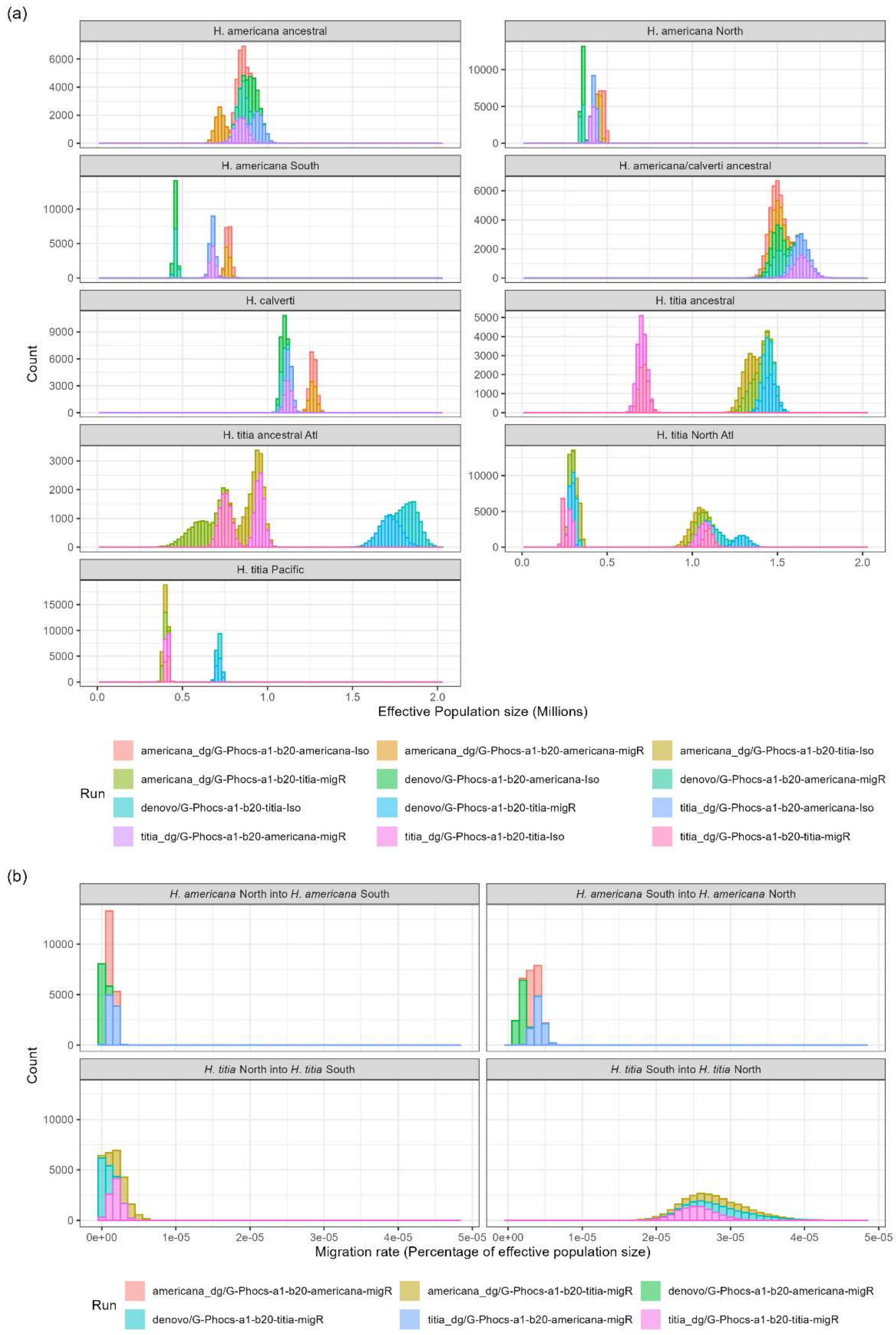

Supplementary Figure 23: Posterior distributions of G-Phocs runs (a) the distribution of effective population sizes for each population, including ancestral population sizes. The legend describes which draft genome (dg) was used (*H. americana*, *H. titia*, or de novo assembly) what the alpha and beta values were set as, which species samples where included, and whether there was complete isolation between lineages (Iso) or migration between the most recently diverged lineages (migR) (b) Posterior of the migration rate as a proportion of effective population size per generation from each model.

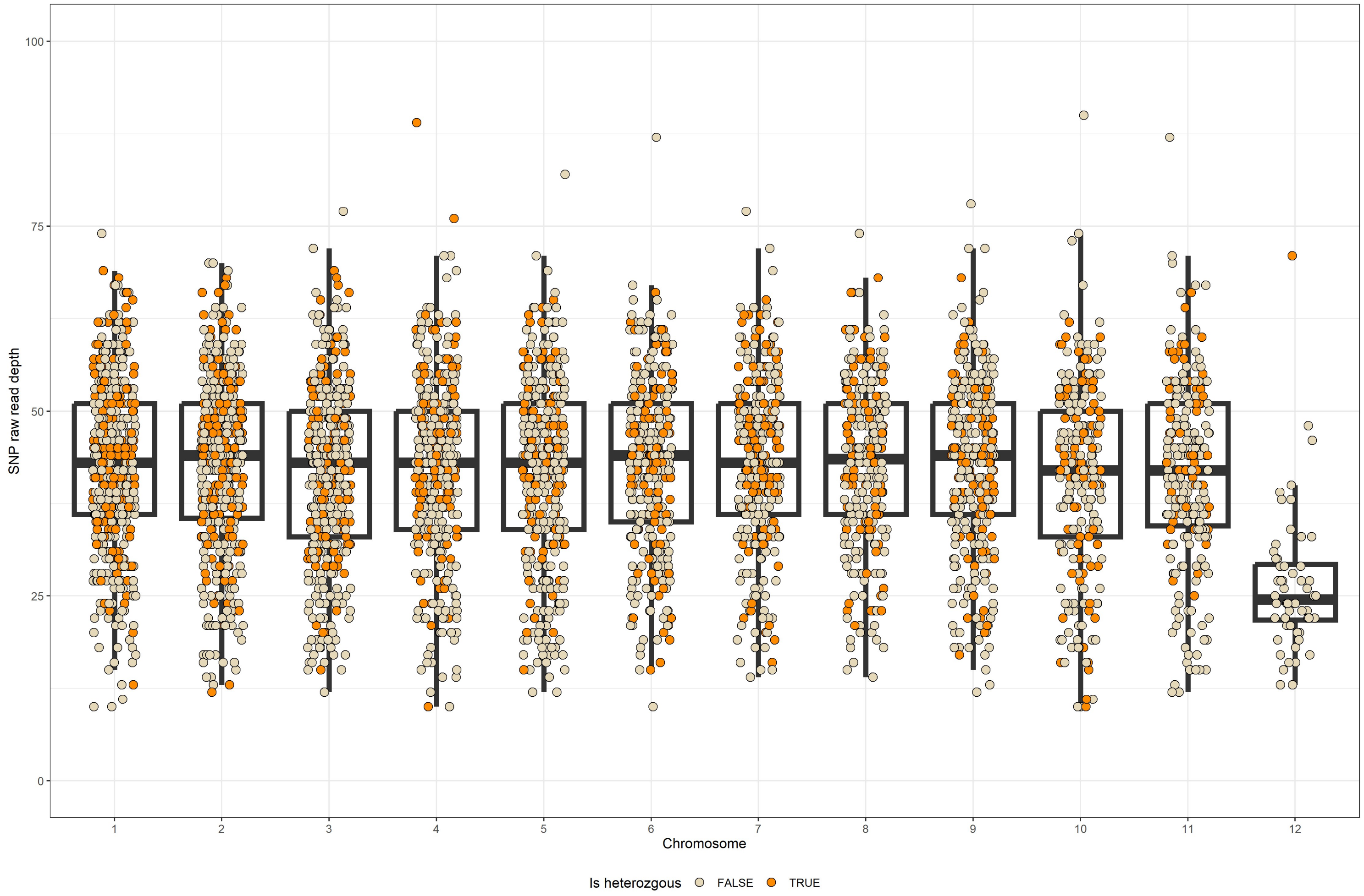

Supplementary Figure 24: All genotyped SNPs from individual CUAJa02 from site CUAJ01, Cuajinicuil, Oaxaca (16° 47'24.00"N, 95°0'36.00"W). Each point marks the raw read number used for SNP calling at that specific site, which chromosome the SNP was mapped to and whether site was heterozygous. Chromosome 12 is the X chromosome. The y axis is restricted to a read coverage of 0 to 100 excluding a small number of high coverage autosomal SNPs.

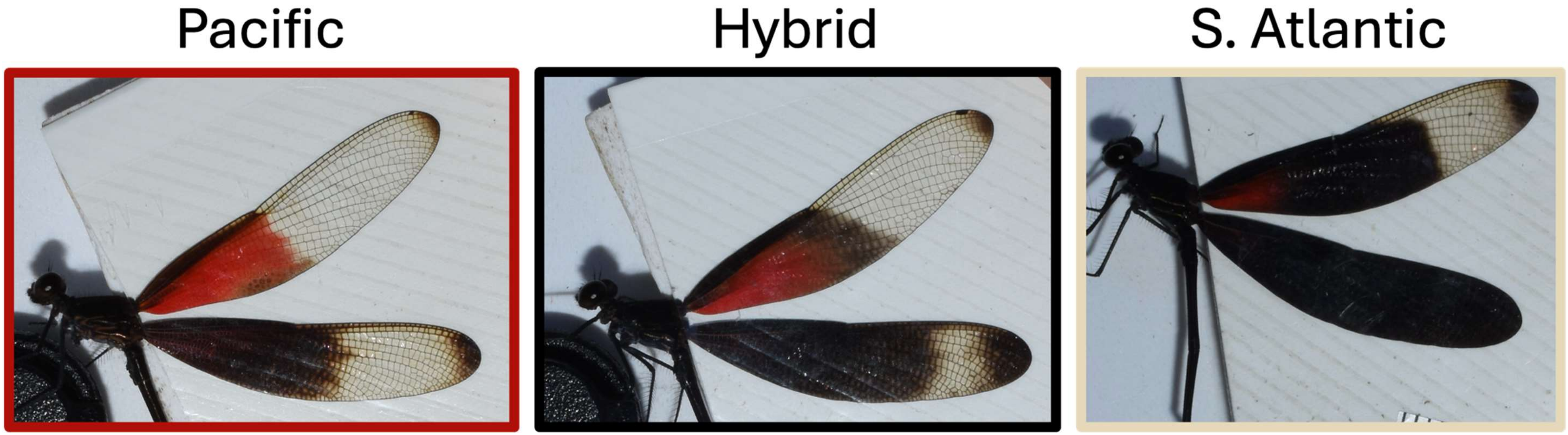

Supplementary Figure 25: Photographs of representative *Hetaerina titia* males around the Isthmus of Tehuantepec (Mexico). From left to right; a typical Pacific phenotype, the hybrid individual CUAJa02 sequenced using ddRAD and genotyped as an F1 hybrid between Pacific and Atlantic lineages of *H. titia*, and a typical South Atlantic phenotype.

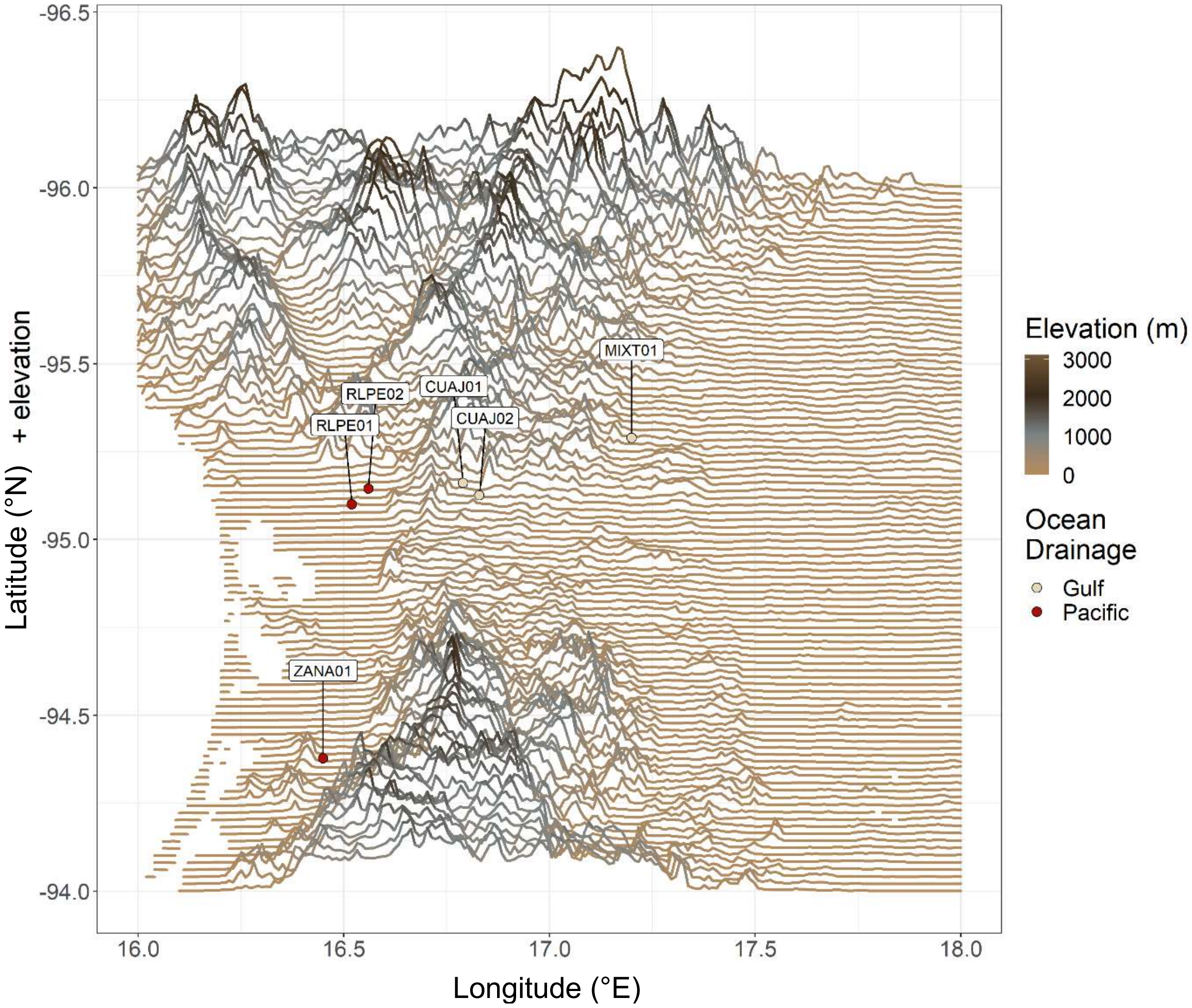

Supplementary Figure 26: Elevational transects running East to West for the Isthmus of Tehuantepec. Each transect is plotted as the longitude of the transect plus the elevation, at each latitude, using the equation: (longitude – (elevation/max_elevation) x 0.4). Sample sites are marked and transformed to be in line with the elevational transects. The F_1 hybrid was collected in an Atlantic river basin at site CUAJ01.

References

Bohling, J. (2020). Evaluating the effect of reference genome divergence on the analysis of empirical RADseq datasets. *Ecology and Evolution*, *10*(14), 7585–7601. https://doi.org/10.1002/ece3.6483

Catchen, J., Hohenlohe, P. A., Bassham, S., Amores, A., & Cresko, W. A. (2013). Stacks: An analysis tool set for population genomics. *Molecular Ecology*, *22*(11), 3124–3140. https://doi.org/10.1111/MEC.12354

DaCosta, J. M., & Sorenson, M. D. (2014). Amplification Biases and Consistent Recovery of Loci in a Double-Digest RAD-seq Protocol. *PLOS ONE*, *9*(9), e106713. https://doi.org/10.1371/journal.pone.0106713

Danecek, P., Bonfield, J. K., Liddle, J., Marshall, J., Ohan, V., Pollard, M. O., Whitwham, A., Keane, T., McCarthy, S. A., Davies, R. M., & Li, H. (2021). Twelve years of SAMtools and BCFtools. *GigaScience*, *10*(2), giab008. https://doi.org/10.1093/gigascience/giab008

de Jong, M. J., de Jong, J. F., Hoelzel, A. R., & Janke, A. (2021). SambaR: An R package for fast, easy and reproducible population-genetic analyses of biallelic SNP data sets. *Molecular Ecology Resources*, *21*(4), 1369–1379. https://doi.org/10.1111/1755-0998.13339

Eaton, D. A. R., & Overcast, I. (2020). ipyrad: Interactive assembly and analysis of RADseq datasets. *Bioinformatics*, *36*(8), 2592–2594. https://doi.org/10.1093/bioinformatics/btz966

Franchini, P., Monné Parera, D., Kautt, A. F., & Meyer, A. (2017). quaddRAD: A new high-multiplexing and PCR duplicate removal ddRAD protocol produces novel evolutionary insights in a nonradiating cichlid lineage. *Molecular Ecology*, *26*(10), 2783–2795. https://doi.org/10.1111/mec.14077

Grether, G. F., Beninde, J., Beraut, E., Chumchim, N., Escalona, M., MacDonald, Z. G., Miller, C., Sahasrabudhe, R., Shedlock, A. M., Toffelmier, E., & Shaffer, H. B. (2023). Reference genome for the American rubyspot damselfly, Hetaerina americana. *Journal of Heredity*, *114*(4), 385–394. https://doi.org/10.1093/jhered/esad031

Keightley, P. D., Ness, R. W., Halligan, D. L., & Haddrill, P. R. (2014). Estimation of the Spontaneous Mutation Rate per Nucleotide Site in a Drosophila melanogaster Full-Sib Family. *Genetics*, *196*(1), 313–320. https://doi.org/10.1534/genetics.113.158758

Li, H. (2011). A statistical framework for SNP calling, mutation discovery, association mapping and population genetical parameter estimation from sequencing data. *Bioinformatics*, *27*(21), 2987–2993. https://doi.org/10.1093/bioinformatics/btr509

Patterson, C. W., Bonillas-Monge, E., Brennan, A., Grether, G. F., Mendoza-Cuenca, L., Tucker, R., Vega-Sánchez, Y. M., & Drury, J. (2023). A chromosome-level genome assembly for the smoky rubyspot damselfly (Hetaerina titia). *Journal of Heredity*, esad070. https://doi.org/10.1093/jhered/esad070

Prates, I., Penna, A., Rodrigues, M. T., & Carnaval, A. C. (2018). Local adaptation in mainland anole lizards: Integrating population history and genome–environment associations. *Ecology and Evolution*, *8*(23), 11932–11944. https://doi.org/10.1002/ece3.4650

Smith, M. L., & Carstens, B. C. (2020). Process-based species delimitation leads to identification of more biologically relevant species. *Evolution*, *74*(2), 216–229. https://doi.org/10.1111/evo.13878

Standring, S., Sánchez-Herrera, M., Guillermo-Ferreira, R., Ware, J. L., Vega-Sánchez, Y. M., Clement, R., Drury, J. P., Grether, G. F., González-Rodríguez, A., Mendoza-Cuenca, L., Bota-Sierra, C. A., & Bybee, S. (2022). Evolution and Biogeographic History of Rubyspot Damselflies (Hetaerininae: Calopterygidae: Odonata). *Diversity*, *14*(9). https://doi.org/10.3390/d14090757

Vega-Sánchez, Y. M., Oyama, K., Mendoza-Cuenca, L. F., Gaytán-Legaria, R., & González-Rodríguez, A. (2024). Genomic differentiation and niche divergence in the Hetaerina americana (Odonata) cryptic species complex. *Molecular Ecology*, *33*(2), e17207. https://doi.org/10.1111/mec.17207
